# Discovery of ((1,2,4-oxadiazol-5-yl)pyrrolidin-3-yl)ureidyl derivatives as selective non-steroidal agonists of the G-protein coupled bile acid receptor-1

**DOI:** 10.1038/s41598-019-38840-z

**Published:** 2019-02-21

**Authors:** Francesco Saverio Di Leva, Carmen Festa, Adriana Carino, Simona De Marino, Silvia Marchianò, Daniele Di Marino, Claudia Finamore, Maria Chiara Monti, Angela Zampella, Stefano Fiorucci, Vittorio Limongelli

**Affiliations:** 1 Department of Pharmacy, University of Naples “Federico II”, via D. Montesano 49, 80131 Naples, Italy; 2 Department of Surgery and Biomedical Sciences, Nuova Facoltà di Medicina, Perugia, Italy; 3 Università della Svizzera italiana (USI), Faculty of Biomedical Sciences, Institute of Computational Science - Center for Computational Medicine in Cardiology, Via G. Buffi 13, CH-6900 Lugano, Switzerland; 4 Department of Pharmacy, University of Salerno, Via Giovanni Paolo II, 132, 84084 Fisciano, Salerno Italy

## Abstract

The G-protein bile acid receptor 1 (GPBAR1) has emerged in the last decade as prominent target for the treatment of metabolic and inflammatory diseases including type 2 diabetes, obesity, and non-alcoholic steatohepatitis. To date numerous bile acid derivatives have been identified as GPBAR1 agonists, however their clinical application is hampered by the lack of selectivity toward the other bile acid receptors. Therefore, non-steroidal GPBAR1 ligands able to selectively activate the receptor are urgently needed. With this aim, we here designed, synthesized and biologically evaluated ((1,2,4-oxadiazol-5-yl)pyrrolidin-3-yl) urea derivatives as novel potent GPBAR1 agonists. Particularly, compounds **9** and **10** induce the mRNA expression of the GPBAR1 target gene pro-glucagon and show high selectivity over the other bile acid receptors FXR, LXRα, LXRβ and PXR, and the related receptors PPARα and PPARγ. Computational studies elucidated the binding mode of **10** to GPBAR1, providing important structural insights for the design of non-steroidal GPBAR1 agonists. The pharmacokinetic properties of **9** and **10** suggest that the ((1,2,4-oxadiazol-5-yl)pyrrolidin-3-yl)ureydil scaffold might be exploited to achieve effective drug candidates to treat GPBAR1 related disorders.

## Introduction

Bile acids (BAs) are amphipathic molecules synthesized from cholesterol in the liver and stored in the gallbladder, from where they are released during digestion to emulsify lipid-soluble nutrients. In addition to their role in diet lipids absorption, bile acids act as signaling molecules involved in the systemic regulation of multiple metabolic pathways. Such endocrine functions are mainly exerted through their interaction with nuclear hormone receptors (NHRs) and cell membrane receptors^1^. Among NRs, the farnesoid X receptor (FXR) plays a major role^2–4^. This receptor is highly expressed in the liver, intestine, kidney, adrenal glands and adipose tissue representing a major regulator of bile acid homeostasis^5^ and glucose, triglyceride and cholesterol metabolism^6–9^. The main example of bile acid cell membrane receptor is the G-protein coupled bile acid receptor 1 (GPBAR1, also known as TGR5 or M-BAR)^10,11^. GPBAR1 is expressed in the brown adipose tissue, muscle, liver, intestine, gallbladder, immune system cells and selected areas of the central nervous system regulating multiple functions^1^. GPBAR1 activation in intestinal enteroendocrine L cells stimulates the transcription of the proglucagon gene expression and the secretion of the incretin GLP-1, thus lowering blood glucose and insulin levels while increasing insulin sensitivity^12–14^. In brown adipose tissue and muscle, GPBAR1 locally stimulates thyroid hormone-mediated thermogenesis, thereby enhancing energy expenditure^15^. In addition, activation of GPBAR1 in macrophages reduces the release of pro-inflammatory cytokines, producing in turn anti-inflammatory effects^16–18^. For all these reasons, the development of GPBAR1 agonists represents an intriguing strategy for the treatment of metabolic disorders like type II diabetes, obesity, inflammatory diseases and some types of cancer^9,19,20^. Starting from the evidence that FXR and GPBAR1 regulate overlapping metabolic functions^21,22^ many efforts were spent for the identification of dual GPBAR1/FXR agonists^23–25^. Nevertheless, these compounds show unwanted side effects due to over-activation of the two receptors^26,27^. As a consequence, most recent studies have focused on the identification of selective ligands towards either FXR or GPBAR1. We and other groups have contributed to this field reporting a number of BA derivatives as selective agonists of these receptors^28–34^. Nevertheless, the clinical use of BA derivatives is hampered by a number of limitations. In particular, they are promiscuous ligands able to activate simultaneously different bile acid receptors such as GPBAR1, FXR, the pregnane X receptor (PXR)^35^ and the liver X receptors (LXR α and β)^36,37^, therefore showing a number of off-target effects. Furthermore, BA derivatives are intrinsically endowed with detergent-like properties forming cytotoxic micellar aggregates^20,38^. These issues have prompted medicinal chemists to design non-BA modulators of these receptors. In particular, the chemical structure of non-BA ligands lacks of the steroidal cycle that is responsible for the promiscuous binding affinity of the BA derivatives to the different BAs receptors. The few reported non-steroidal GPBAR1 ligands show promising results against type 2 diabetes and other GPBAR1 related disorders^39–43^. Therefore, the discovery of novel selective non-steroidal GPBAR1 agonists has become in great demand. With this aim, we performed a structure-based drug design study, using the 3D model of GPBAR1 that we have recently reported^24^, which led to the identification of a new series of non-steroidal selective GPBAR1 agonists. These compounds have a ((1,2,4-oxadiazol-5-yl)pyrrolidin-3-yl)ureidyl core that represents a new chemical scaffold for GPBAR1 ligands if compared with the previously reported compounds^9,20,22^. Here, we show that this scaffold is crucial for the binding interaction to the orthosteric site residues of the receptor. Using transactivation assays, we demonstrate the ability of the new series of compounds to activate selectively GPBAR1 over the other BAs receptors as FXR, LXRα, LXRβ and PXR, and the related receptors PPARα and PPARγ. The most potent derivatives, **9** and **10**, demonstrate to induce the expression of mRNA of the GPBAR1 target gene pro-glucagon, with compound **10** showing the same efficacy of the endogenous agonist TLCA^10,11^. We have elucidated the binding mode of the most potent compound of the series (**10**) combining docking calculations and molecular dynamics simulations, showing that the residues Glu169, Asn93 and Tyr240 play a leading role in the ligand recognition. While the same residues are known to be involved in the binding of BA derivatives^33,34,44,45^, this is an unprecedented structural insight for non-steroidal GPBAR1 agonists that paves the way to following lead-optimization and drug design studies. The high GPBAR1 activity efficacy and the optimal pharmacokinetic profile of the new compounds place the ((1,2,4-oxadiazol-5-yl)pyrrolidin-3-yl)ureidyl scaffold in an optimal fashion for further investigations to achieve drug candidates to treat GPBAR1-related metabolic disorders.

## Results

### Design and Synthesis

**Figure 1:**
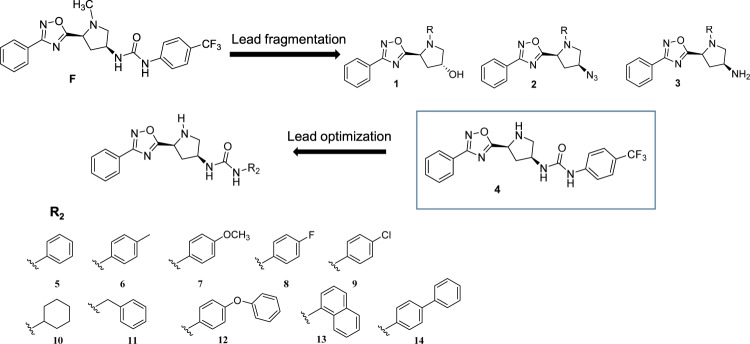
Compound F from VS and derivatives **1**–**14** identified in this study as a new chemotype of GPBAR1 agonists.

In the very first stage of our research, we performed a docking-based virtual screening (VS) study, using the 3D model of GPBAR1 that we have recently reported^24^, to identify new potential GPBAR1 ligands. This procedure led to the identification of eleven compounds (Compounds A-L in Supplementary Fig. S1) that were purchased from AnalytiCon (http://www.ac-discovery.com). These compounds underwent a thorough characterization of their purity and chemical structures. This investigation showed that the structure of one of the purchased ligands did not correspond to that declared by the vendor and evaluated by our calculations. Specifically, NMR spectroscopy studies revealed the presence of a *meta*-CF_3_ phenyl ring instead of the *para*-CF_3_-phenyl moiety (see Supplementary Figs S2 and S3) in compound F. This discovery prompted us to synthesize *ab initio* the *para*-CF_3_ phenyl analogue (Compound F from VS, Fig. 1). Unfortunately, transactivation assay on GPBAR1 revealed that none of compounds A-L from VS was endowed with remarkable activity when compared to TLCA, the endogenous GPBAR1 agonist (Supplementary Fig. S4). Nevertheless, we noticed that compound F as well as several of the other identified putative GPBAR1 ligands from VS share the 3,5-disubstituted 1,2,4-oxadiazole moiety (compounds F, G, H, J), which resembles previously reported GPBAR1 agonists^20^. Furthermore, the 1,2,4-oxadiazole ring is a common privileged scaffold that has emerged as a core structural unit of various bioactive compounds. This heterocycle can indeed act as bioisostere for both amide and carboxylic acid  moieties, improving the pharmacokinetic and pharmacodynamic profile, enhancing selectivity, fixing conformations and modulating polarity of the drug candidates. After the set up of the total synthesis of compound F, we tested using transactivation assays the simplified analogues in our hands (compounds **1**–**4** in Fig. 1) including the *N*-demethylated analogue of compound F, compound **4**. The latter showed moderate GPBAR1 agonist activity that prompted us to explore further the chemical space around the (1,2,4-oxadiazol-5-yl)pyrrolidin-3-yl scaffold in a lead optimization study. Finally, this process led to the identification of a new class of GPBAR1 agonists.

As regards the organic synthesis, 1,2,4-oxadiazoles **1**–**14** were prepared in a convergent synthesis from benzonitrile **15** and (3*R*)-hydroxy-Boc-*L*-proline. Briefly, treatment of **15** with hydroxylamine hydrochloride afforded the intermediate amidoxime **16** (quantitative yield), that was coupled with (3*R*)-hydroxy-Boc-*L*-proline by using (2-(1H-benzotriazol-1-yl)-1,1,3,3-tetramethyluronium hexafluorophosphate (HBTU) as coupling agent, yielding to the formation of compound **17** (60% yield). Mesylation on **17** followed by treatment with NaN_3_ furnished the intermediate **18**. The reduction of the azido group with NH_4_Cl/Zn powder gives the amino derivative **19**, as a key intermediate in the preparation of compound F and compounds **4**–**14**.

**Figure 2:**
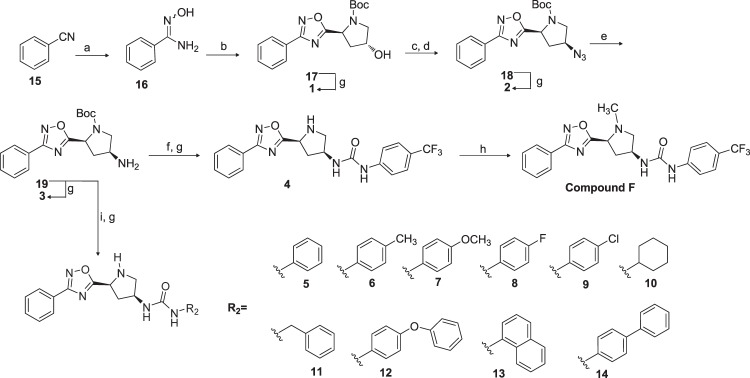
Preparation of compound F from VS, its simplified derivatives **1**–**4** and optimized GPBAR1 agonists **5**–**14**. Reagents and conditions: (a) NH_2_OH·HCl, K_2_CO_3_, CH_3_OH reflux, quantitative yield; (b) (3*R*)-hydroxy-Boc-*L*-proline, DIPEA, HBTU in DMF, 80 °C, 60%; (c) MsCl, TEA, −10 °C, 2 h, quantitative yield; (d) NaN_3_ in DMSO, 150 °C, 4 h, 75%; (e) NH_4_Cl, Zn, CH_3_OH:H_2_O (1:0.1), 61%; (f) 4-(trifluoromethyl)phenyl isocyanate, CH_2_Cl_2_, 0 °C, 84%; (g) TFA, 2 h, quantitative yield; (h) HCHO, HCOOH, 80–90 °C overnight, 56%; (i) R_2_NCO, CH_2_Cl_2_, 0 °C, 74%.

First, treatment of **19** with 4-(trifluoromethyl)phenylisocyanate followed by acid removal of N-protecting group afforded compound **4** whose *N*-methylation by reductive amination furnished compound F in 56% yield (Fig. 2). Then treatment of **19** with differentiated isocyanates, followed by acid removal of the *N*-Boc protecting group, afforded compounds **5–14**. Finally, for lead fragmentation process, little amounts of intermediates **17**, **18** and **19** have been Boc-deprotected affording compounds **1**, **2** and **3** respectively.

### Pharmacological Evaluation

**Figure 3:**
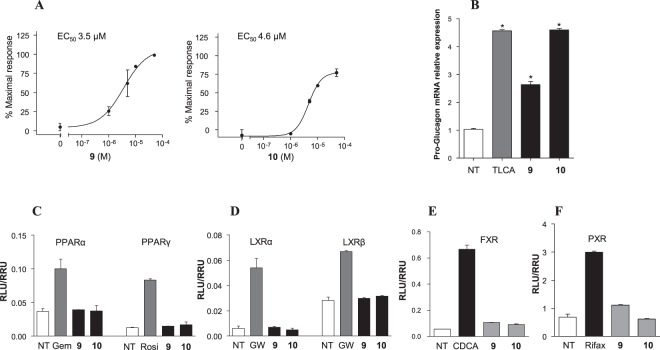
(**A**) Dose response curves of compounds **9** and **10** (concentrations ranging from 1 to 50 µM) in luciferase reporter assay. (**B**) Real-time PCR analysis of mRNA expression of Pro-glucagon in GLUTag cells treated with compounds **9** and **10** at 10 μM, and TLCA used as a positive control (10 μM). Values are normalized to GAPDH and are expressed relative to those of not treated cells (NT) which are arbitrarily settled to 1. The relative mRNA expression is expressed as 2^(−ΔΔCt)^. (**C**) Specificity of compounds **9** and **10** at 10 μM dose in luciferase reporter assays versus PPARα and PPARγ using Gemfibrozil (GEM, 10 μM) and Rosiglitazone (ROSI, 100 nM) as positive controls respectively. (**D**) Specificity of compounds **9** and **10** at 10 μM in luciferase reporter assays versus LXRα and LXRβ using GW3965 (GW, 10 μM) as positive control. (**E**) Specificity of compounds **9** and **10** at 10 μM in luciferase reporter assays versus FXR using CDCA (10 μM) as positive control; (**F**) Specificity of compounds **9** and **10** at 10 μM in luciferase reporter assays versus PXR using Rifaximin (Rifax, 10 μM) as positive control.

The activity of compounds **1**–**14** on GPBAR1 was evaluated in a luciferase reporter assay using HEK-293T cells transfected with GPBAR1. The efficacy of tested compounds (Table 1) was measured using taurolithocholic acid (TLCA) as GPBAR1 reference compound^10,11^. Each compound was tested at the concentration of 10 μM and transactivation activity of TLCA on a cAMP responsive element (CRE) (i.e. TGR5/GPBAR1) was considered equal to 100%. As shown in Table 1, the efficacy of compounds **1–14** was in 58–121% range, with compounds **9** (EC_50_ 3.5 μM, 115% efficacy) and **10** (EC_50_ 4.6 μM; 121% efficacy) showing the best efficacy/potency match (Table 1 and Fig. 3, panel A). Moreover, the agonism of **9** and **10** was further assessed by RT-PCR experiments in which each of two compounds was effective in inducing the expression of pro-glucagon mRNA in GLUTag cells, an intestinal endocrine cell line (Fig. 3, panel B). Remarkably, **10** shows the same efficacy of TLCA in inducing pro-glucagon mRNA expression. Thus, **9** and **10** (Table 1) turned out to be the most promising derivatives considering their low EC_50_ values and high activity efficacy. To further characterize the pharmacological properties of these compounds, we expanded their *in vitro* profile to common off-targets for bile acid receptor ligands. As showed in Fig. 3, panels C–F, **9** and **10** (10 μM) were unable to induce PPARα/PPARγ, LXRα/LXRβ as well as FXR and PXR transactivation on HepG2 cells.

**Table 1:** Efficacy data resulted from comparison of the maximal transactivation of CRE (cAMP responsive element) caused by each compound (10 μM) with that of TLCA (10 μM).

N°	Compound	Efficacy (% vs TLCA)
**1**		72%
**2**		58%
**3**		74%

**R**_**2**_		
**4**	4-CF_3_-Ph	68%
**5**	Ph	79%
**6**	4-Me-Ph	65%
**7**	4-OMe	81%
**8**	4-F	96%
**9**	4-Cl	115%
**10**	C_6_H_11_	121%
**11**	CH_2_Ph	78%
**12**	4-OPh-Ph	85%
**13**	Naphtyl	95%
**14**	Biphenyl	95%

Data are calculated from the same experiment conducted in triplicate.

### I*n vitro* pharmacokinetics characterization

In order to assess the drug-likeness of **9** and **10**, we evaluated some of their physiochemical properties such as the partition coefficient (clogP), the aqueous solubility and the *in vitro* rat liver microsomal stability (Table 2). Remarkably, **9** and **10** show aqueous solubility of 42 μg/mL (110 μM) and 70 µg/mL (198 µM), respectively, and a moderate lipophilicity (**9**, logD = 1.47; **10**, logD = 1.25). Notably, both compounds offer the possibility to improve their solubility and polarity by a salt formation via the amine group and, according to Lipinski’s Rule of Five, exhibit a clogP value between 1 and 2, which is favorable for orally administered drugs. Finally, **9** and **10** are characterized by a medium clearance (Cl) and a moderate half-life (t_1/2_) (Table 2), with 42% and 40% of **9** and **10** remaining 40 min after treatment, respectively, and both parameters are indicative of their good metabolic stability *in vitro*.

**Table 2:** *In vitro* pharmacokinetics for selected oxadiazole derivatives **9** and **10**.

Compound	R_2_	Aqueous solubility (μM)	Log D	CI_int_ (μL/min/mg protein)	t_½_ (min)

**9**		110	1.47	67	35
**10**		198	1.25	76	30

### Molecular Modeling

In order to elucidate the binding mode of the newly synthesized derivatives to GPBAR1, molecular modeling studies were carried out on the most promising derivative of the series, **10**. First, molecular docking calculations were performed on **10** in the 3D homology model of GPBAR1, which we originally built in 2014^24^ and validated in the following years with a number of successful drug design studies^33,34,46,47^. Among the binding modes predicted by docking, only two poses interact with Glu169 of GPBAR1 (Supplementary Fig. S5), which is known to be involved in the binding of agonists and receptor activation^24,44,45^. Therefore, these two poses were further investigated through more accurate calculations. In particular, each pose underwent to 150 ns MD simulations in explicit solvent and membrane not only to fully take into account the receptor flexibility and the solvent effect but also to evaluate the energetics and structural stability of the two binding modes.

**Figure 4:**
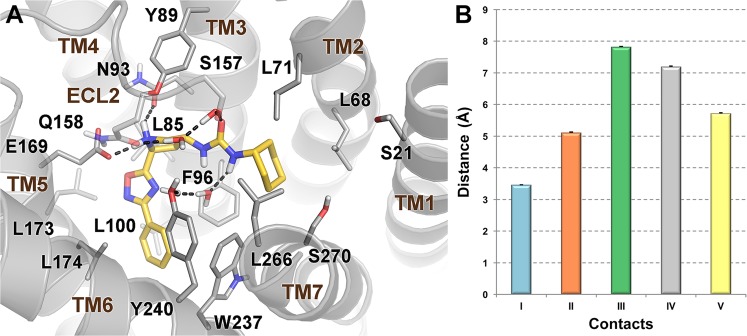
(**A**) Binding mode of **10** (yellow sticks) to GPBAR1 (gray cartoon). Amino acids essential for ligand binding are depicted as sticks. Polar contacts are shown as dashed black lines. ECL1, residues 145–156 in ECL2, ECL3 and nonpolar hydrogens are undisplayed for clarity. (**B**) Interatomic distances (mean ± S.E.M.) representative of the main ligand/receptor contacts observed along the last 100 ns of MD simulations: (I) pyrrolidinyl ring (N)/Glu169 (Cδ), cyan bar; (II) pyrrolidinyl ring (N)/Asn93 (Cγ), orange bar; (III) phenyl (centroid)/Tyr240 (centroid of the phenolic ring), green bar; (IV) ureidic moiety (C)/Tyr (OH), gray bar; (V) cyclohexyl (centroid)/Leu266 (Cγ), yellow bar.

The MD calculations show that only one of the two docking poses is stable over the simulation time, as shown by the values of the root mean square deviation (rmsd) computed for the ligand heavy atoms (Supplementary Fig. S6). In the energetically most stable pose (Fig. 4A), the ligand pyrrolidinyl ring engages a salt bridge with the Glu169 side chain on transmembrane helix (TM) 5 and a H-bond with Asn93 on TM3, while the phenyl-oxadiazolyl moiety deepens in the lipophilic pocket formed by the side chains of residues such as Leu85, Phe96, Leu100, Leu173, Leu174, and Trp237 and Tyr240. On the other side, the ligand cyclohexyl ring occupies the small hydrophobic cleft defined by Leu68 and Leu71 on TM2 and Phe96 on TM3, which are involved in the binding of some BA derivatives in our recent publication^46^, and it also contacts the side chain of Leu266. Finally, the ureidic group of **10** forms water mediated interactions with Ser157, Gln158 on the extracellular loop (ECL) 2, and Tyr240 on TM6. Our results indicate that along the MD simulation the ligand forms stable interactions with the residues Asn93, Glu169, Tyr240 and Leu266 (Fig. 4B). It is interesting to note that the same residues are involved in the binding of BA derivatives to GPBAR1 and showed to be relevant for its activation^24,33,34,44,45^. For the first time here we report that Asn93, Glu169, Tyr240 work as anchor point also for non-steroidal ligands and should be targeted in drug design of new GPBAR1 agonists.

Finally, we note that a few of the ligand/receptor contacts predicted by MD were not found by docking calculations. This is due to the fact that the docking program neglects the presence of explicit water molecules and treats the protein as rigid, leading to a less accurate description of the ligand/receptor binding process. Based on our results, we suggest using more rigorous calculations that include an explicit solvent model and full receptor flexibility to disclose with accuracy the binding mode of non-steroidal GPBAR1 agonists.

## Discussion

Among the BA receptors, GPBAR1 is considered a prominent target for the treatment of metabolic and inflammatory disorders. A selective modulation of this receptor is sought to avoid side effects due to over-activation of the signaling cascades controlled by other BA receptors. However, this task is challenging since BA receptors share similar structural requisites for ligand binding. In addition, most of the known ligands are BA derivatives that present the steroidal scaffold responsible for the promiscuous binding to the different BA receptors. In this background, the discovery of non-steroidal GPBAR1 agonists is of great demand, offering the opportunity to achieve a potent and selective modulation of the receptor.

In the present work we have pursued this goal combining computational, organic synthesis and pharmacological techniques that led to the identification of ((1,2,4-oxadiazol-5-yl)pyrrolidin-3-yl)ureidyl derivatives as new class of non-steroidal selective GPBAR1 agonists. The pharmacological characterization of these compounds shows high activity efficacy and selectivity towards GPBAR1 over the other BAs receptors as FXR, LXRα, LXRβ and PXR, and the related receptors PPARα and PPARγ. The most potent compounds of the series, **9** and **10**, demonstrated to induce the expression of mRNA of the GPBAR1 target gene pro-glucagon in GLUTag cells, with compound **10** showing the same efficacy of TLCA used as GPBAR1 agonist reference compound. We have performed docking calculations and molecular dynamics simulations to disclose the binding mode of the most potent compound of the series, **10**, showing that the ligand engages stable interactions with the receptor amino acids Asn93, Glu169, Tyr240 and Leu266. The same residues are involved in the binding of BA derivatives to GPBAR1^24,33,34,44,45^, however this is the first time that they are found to be crucial also for the recognition of non-steroidal ligands. This unprecedented structural insight on ligand/receptor binding is valuable to guide further drug design and lead optimization studies. Finally, the discovered ligands display optimal pharmacokinetic features such as suitable aqueous solubility and high microsomal stability. All together our findings prompt further investigations on the ((1,2,4-oxadiazol-5-yl)pyrrolidin-3-yl)ureidyl scaffold to achieve drug candidates for the treatment of GPBAR1 related disorders.

## Methods

### Chemistry

NMR spectra were performed on Varian Inova 400, 500 and 700 spectrometers (400 MHz, 500 and 700 MHz for ^1^H and 100, 125 and 175 MHz for ^13^C, respectively). Residual signals of solvent were used as internal standards (δ_H_ 3.31 ppm and δ_C_ 49.0 ppm for CD_3_OD, δ_H_ 7.26 ppm and δ_C_ 77.0 ppm for CDCl_3_). Data for ^1^H NMR spectra are reported as chemical shift (δ, ppm), integration, molteplicity, coupling costant (Hz). Molteplicity of signals is given as singlet (s), broad singlet (br s), doplet (d), triplet (t), multiplet (m). Micromass Q-TOF mass spetcrometer was used to record high-resolution ESI-MS spectra.

The solvents and reagents were purchased from Sigma-Aldrich in ≥98% purity. All reactions were carried out under Ar atmosphere. Dichloromethane, ethyl ether and triethylamine were distilled from calcium hydride; methanol was dried from magnesium methoxide as previously reported^31^. The reaction were monitored with Alugram silica gel TLC plate, under UV light (254 and 365 nm). Column chromatography was performed on silica gel (200–400 mesh, from Macherey-Nagel Company). HPLC separations were performed using a Water Model 510 pump, Waters Rheodine injector and Differential Refractormeter Model 401. Analytical HPLC analysis was used also to determine the purity of compounds (>95%), using a Nucleodur Sphinx RP column (Macherey-Nagel Company, 5 µm; 4.6 mm i.d.x250 mm, flow rate 1 mL/min) eluiting with the conditions reported for each individual compound in the supporting information.

### Cell culture

HEK-293T and GLUTag cells were cultured and maintained at 37 °C and 5% CO_2_ in D-MEM additioned with 10% FBS, 1% glutamine and 1% penicillin/streptomycin.

### Luciferase reporter gene assay and dose-response curves

Transactivation assay on HEK-293T cells was performed to investigate GPBAR1 activation, using compound **1**–**14** and TLCA as positive control at 10 µM, as previously reported^47^. EC_50_ of compounds **9** and **10** was evaluated treating HEK-293T cells transfected with increasing concentrations of two compounds (range from 1 to 50 µM).

The specificity of compounds **9** and **10** versus PPARα, PPARγ, LXRα, LXRβ, FXR and PXR was determined in luciferase reporter gene assays as previously reported^29,31^.

### RNA isolation and RT-PCR

Evaluation of proglucagon mRNA expression was performed in RT-PCR on GLUTag cells (1 × 10^6^ cells/well in a 6 well plate) as previously reported^34,47^. The relative mRNA expression was calculated and expressed as 2^−(ΔΔCt)^. Forward and reverse primer sequences were the following: mouse GAPDH, ctgagtatgtcgtggagtctac and gttggtggtgcaggatgcattg; mouse Pro-glucagon, tgaagacaaacgccactcac and caatgttgttccggttcctc.

### LC-MS/MS ADME Methods

Chromatography was performed using a HPLC–MS system Q-ToF Premiere instrument (Waters, Co.) equipped with an ESI source and Waters pump systems. The mixture was separated on a Luna 5 µm C8(2) 100 °A 150 × 2 mm from Phenomenex. The mobile phase consisted of 0.2% formic acid (FA) in water as solvent A and 0.2% FA in acetonitrile as solvent B at a flow rate of 200 μL/min. The gradient was as follows: 0–2 min (75% A and 25% B), 2–20 min (5% A and 95% B), 20–30 min (75% A and 25% B). The detection of analytes was achieved by ESI in the positive mode with the appropriate MRM transition or by UV system at 220 nm.

### Solubility Measurements

Ten μL of a 10 mM solution in DMSO of the compound was diluted either in 490 μL of PBS pH 7.4 or in the organic solvent MeOH (in triplicate). The tubes were gently shaken 24 h at room temperature, then centrifuged for 5 min at 4000 rpm. Ten μL of sample was diluted in 490 μL of MeOH. The solubility is determined by the ratio of mass signal area PBS/organic solvent.

### LogD Measurements

Ten μL of a 10 mM solution in DMSO of the compound was diluted in 490 μL of a 1/1 octanol/PBS mixture at pH 7.4. The mixture was gently shaken for 2 h at room temperature. Ten microliters of each phase was diluted in 490 μL of MeOH and analyzed by LC-MS/MS. Each compound was tested in triplicate. Log D was determined as the logarithm of the ratio of concentration of product in octanol and PBS, determined by mass signals.

### Microsomal Stability

Male mouse (CD-1) liver microsomes (Sigma-Aldrich) were used. All incubations were performed in duplicate in a shaking water bath at 37 °C. The incubation mixtures contained 1 μM compound with 1% DMSO used as a vehicle, mouse liver microsomes (0.3 mg of microsomal protein per mL), 5 mM MgCl_2_, 1 mM NADP, 5 mM glucose 6-phosphate, 0.4 U·mL^-^^1^ glucose 6-phosphate dehydrogenase, and 50 mM potassium phosphate buffer (pH 7.4) in a final volume of 0.5 mL. Aliquots were removed at 0, 5, 10, 20, 30, and 40 min after microsome addition and the reaction was stopped by adding 200 μL of ice-cold acetonitrile. After 2 h, the samples were centrifuged for 10 min at 10000 rpm, and the supernatants were transferred in matrix tubes for LC-MS/MS analysis. Propranolol, known as a high hepatic clearance drug in rodents, was used as a quality-control compound for the microsomal incubations. The slope of the linear regression of the curve obtained reporting the natural logarithm of compound area versus incubation time (−k) was used in the conversion to *in vitro* t_1/2_ values by t_1/2_ = −ln(2)/k. *In vitro* intrinsic clearance (Cl_int_ expressed as μL/min/mg) was calculated according to the following formula: Cl_int_ = volume of reaction (μL)/t_1/2_ (min)/protein of liver microsomes (mg).

### Molecular docking

The Glide software package^48^ was used to perform molecular docking calculations of **10** in the three-dimensional model of hGPBAR1^24^. The ligand tridimensional structure was generated through the Maestro Build Panel^49^ and prepared for docking using LigPrep^50^. The ligand protonation states at pH 7.4 +/− 1.0 were assigned using Epik^51,52^. The GPBAR1 model was instead prepared as described in our previous paper^33^. For grid generation a box of 25 × 25 × 25 Å centered on the binding cavity previously identified for bile acid ligands^24^ was created. The Glide standard precision (SP) scoring function^53,54^ was used to score and rank the predicted binding poses. The twenty best ranked docking solutions were visually inspected.

### Molecular dynamics

The predicted docking complexes were embedded in a 1-Palmitoyl-2-oleoylphosphatidylcholine (POPC) phospholipids bilayer following a previously published protocol^24^. Each complex was then solvated according to the TIP3 water model through the solvation module of VMD 1.9.3. The *ff14SB*^55^, *lipid14*^56^ and *gaff *^57^ Amber force fields were used to parameterize the protein, the lipids and the ligand, respectively. The addition of 12 Cl^−^ ions ensured neutrality. Missing ligand charges and parameters were derived by means of the Antechamber program^58^, as described in a previous paper^24^. Each complex was then submitted to 150 ns MD simulations with NAMD 2.11^59^, using the setup described in a previous publication^24^.

All of the pictures were rendered using PyMOL (www.pymol.org).

## Supplementary information

revised supplementary information

supplementary information
