# Supplementary material for: Discovery of ((1,2,4-oxadiazol-5-yl)pyrrolidin-3-yl)ureidyl derivatives as selective non-steroidal agonists of the G-protein coupled bile acid receptor-1
Source: Sci Rep. 2019 Feb 21;9:2504. doi: 10.1038/s41598-019-38840-z (PMC6385358; doi:10.1038/s41598-019-38840-z)
Supplement: Supplementary file 2 — supplementary information [file 41598_2019_38840_MOESM2_ESM.docx]

**SUPPLEMENTARY INFORMATION**

**Discovery of ((1,2,4-oxadiazol-5-yl)pyrrolidin-3-yl)ureidyl derivatives as selective non-steroidal agonists of the G-protein coupled bile acid receptor-1**

Francesco Saverio Di Leva^1§^, Carmen Festa^1§^, Adriana Carino,^2^ Simona De Marino,^1^ Silvia Marchianò,^2^ Daniele Di Marino^3^, Claudia Finamore,^1^ Maria Chiara Monti,^4^ Angela Zampella^1^, Stefano Fiorucci^2^ and Vittorio Limongelli^1,3*^

^1^ Department of Pharmacy, University of Naples "Federico II", via D. Montesano 49, 80131 Naples, Italy.

^2^ Department of Surgery and Biomedical Sciences, Nuova Facoltà di Medicina, Perugia, Italy

^3^ Università della Svizzera Italiana (USI), Faculty of Biomedical Sciences, Institute of Computational Science - Center for Computational Medicine in Cardiology, Via G. Buffi 13, CH-6900 Lugano, Switzerland.

^4^ Department of Pharmacy, University of Salerno, Via Giovanni Paolo II, 132, 84084, Fisciano, Salerno, Italy.

^§^ These authors equally contributed to this work.

*Corresponding Author: Prof. Dr. Vittorio Limongelli; e-mail: [vittoriolimongelli@gmail.com](mailto:vittoriolimongelli@gmail.com)

**Table of Contents Page**

1. **Supplementary Figure S1.** Compounds A-L from VS S3
2. **Supplementary Figure S2.** ^1^H NMR (700 MHz, CD_3_OD) of purchased compound F S4
3. **Supplementary Figure S3.** ^1^H NMR (700 MHz, CD_3_OD) of prepared compound F S5
4. **Supplementary Figure S4**. Transactivation assay on compounds A-L from VS S6
5. **Supplementary Figure S5.** Docking poses of **10** S8
6. **Supplementary Figure S6.** Ligand heavy atoms rmsd along the MD simulations S8
7. **Supplementary Figure S7.** Rmsd of the GPBAR1 TMHs Cα along the MD simulations S8
8. **Synthetic Procedures for compounds 1-14 and compound F** S9
9. **Supplementary Figure S8**. ^1^H NMR (400 MHz, CD_3_OD) of compound **1** S16
10. **Supplementary Figure S9**. ^1^H NMR (400 MHz, CD_3_OD) of compound **2** S17
11. **Supplementary Figure S10**. ^1^H NMR (400 MHz, CD_3_OD) of compound **3** S18
12. **Supplementary Figure S11**. ^1^H NMR (500 MHz, CD_3_OD) of compound **4** S19
13. **Supplementary Figure S12**. ^1^H NMR (400 MHz, CD_3_OD) of compound **5** S20
14. **Supplementary Figure S13**. ^1^H NMR (400 MHz, CD_3_OD) of compound **6** S21
15. **Supplementary Figure S14**. ^1^H NMR (400 MHz, CD_3_OD) of compound **7** S22
16. **Supplementary Figure S15**. ^1^H NMR (400 MHz, CD_3_OD) of compound **8** S23
17. **Supplementary Figure S16**. ^1^H NMR (400 MHz, CD_3_OD) of compound **9** S24
18. **Supplementary Figure S17**. ^1^H NMR (500 MHz, CD_3_OD) of compound **10** S25
19. **Supplementary Figure S18**. ^1^H NMR (400 MHz, CD_3_OD) of compound **11** S26
20. **Supplementary Figure S19**. ^1^H NMR (400 MHz, CD_3_OD) of compound **12** S27
21. **Supplementary Figure S20**. ^1^H NMR (400 MHz, CD_3_OD) of compound **13** S28
22. **Supplementary Figure S21**. ^1^H NMR (500 MHz, CD_3_OD) of compound **14** S29

**Supplementary Figure S1.** Compounds A-L from VS


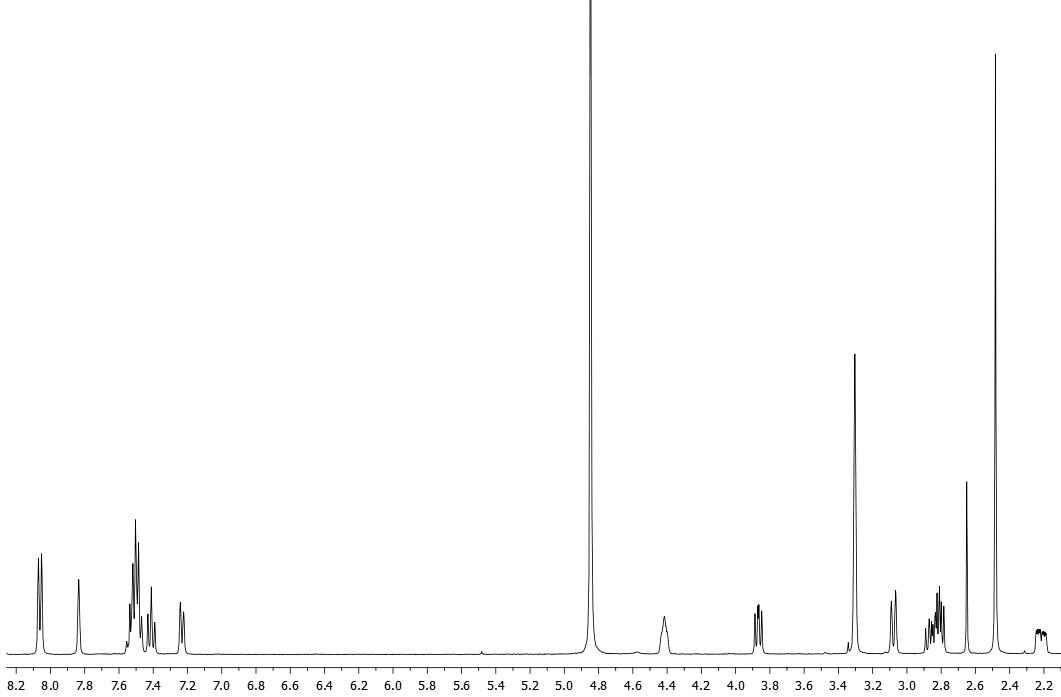


**Supplementary Figure S2.** ^1^H NMR (700 MHz, CD_3_OD) of purchased compound F


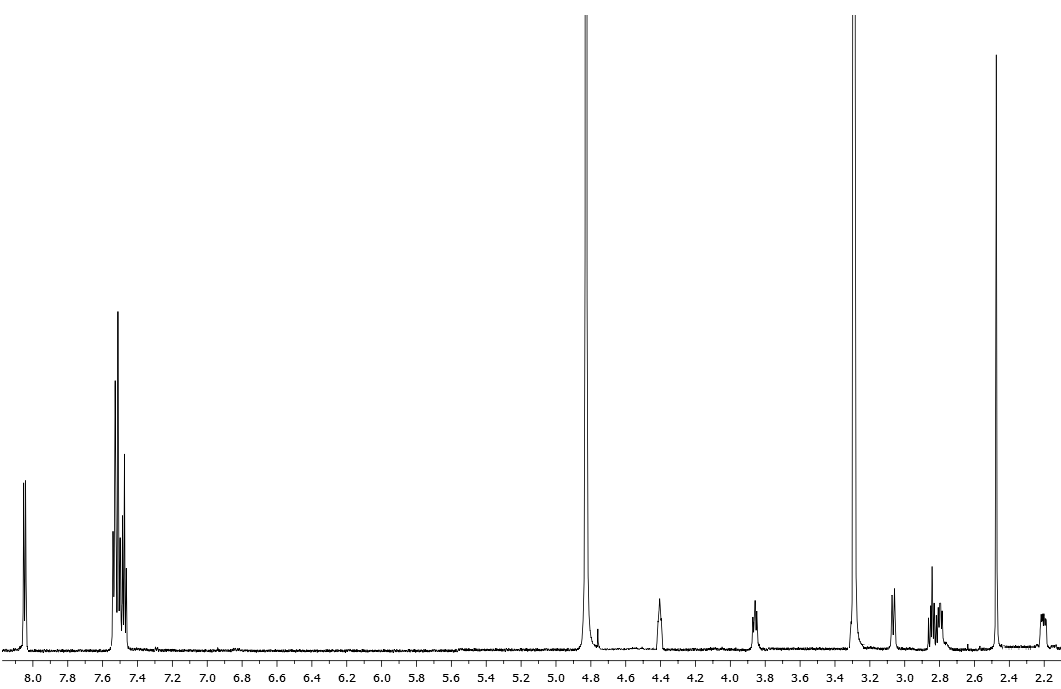


**Supplementary Figure S3.** ^1^H NMR (700 MHz, CD_3_OD) of prepared compound F

**Supplementary Figure S4**. HEK-293T cells were transiently transfected with GPBAR1 and a reporter gene containing a cAMP responsive element in front of the luciferase gene. Cells were stimulated with compounds A-L (10 μM). TLCA (10 μM) was used as positive control. Luciferase activity served as a measure of the rise in intracellular cAMP following activation of GPBAR1. In all panels, results are expressed as mean ± standard error. ^∗^p < 0.05 *versus* not treated cells (NT).


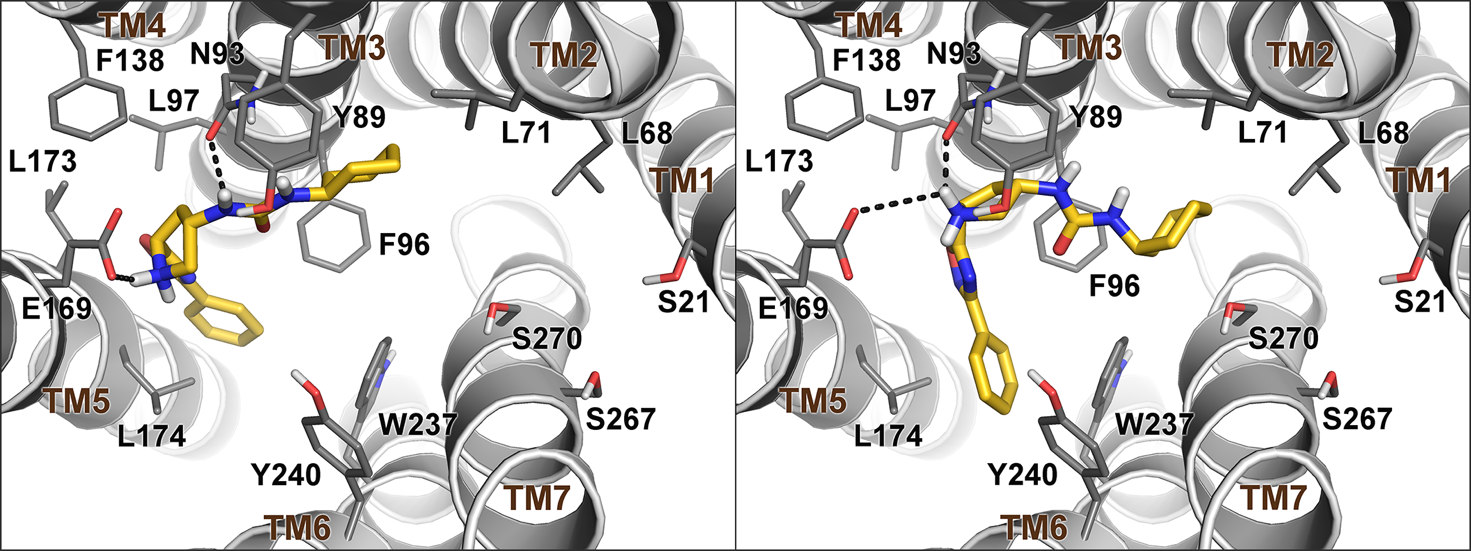


**Supplementary Figure S5.** Binding poses A (left) and B (right) of **10** (yellow sticks) at GPBAR1 (gray cartoons) as predicted by docking calculations. Amino acids important for ligand binding are shown as sticks. Polar contacts are shown as dashed black lines. Extracellular loops and nonpolar hydrogens are omitted for clarity.


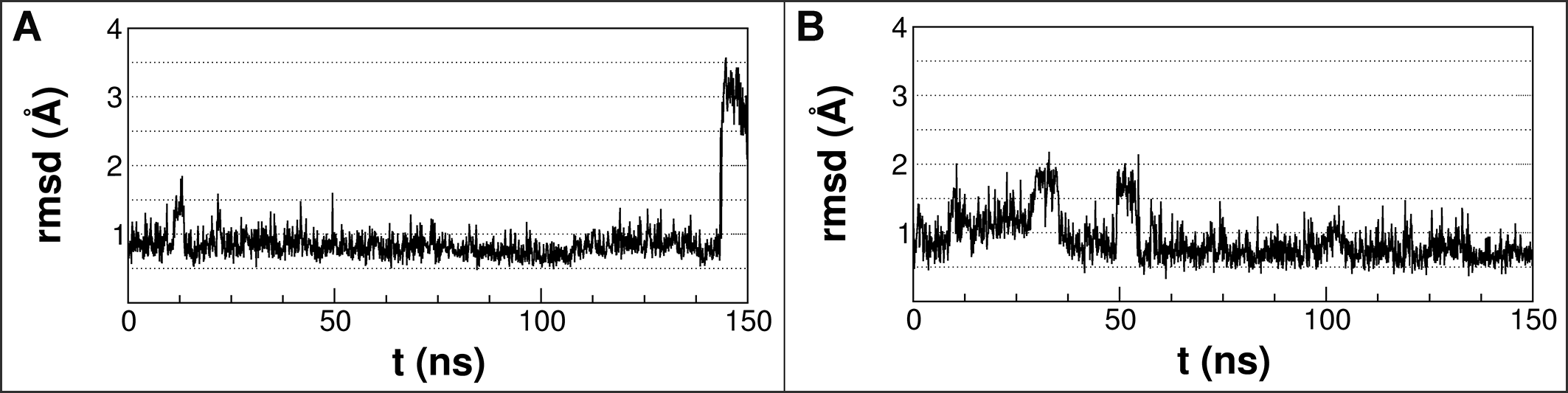


**Supplementary Figure S6.** Average rmsd of the **10** heavy atoms along the MD simulations on the docking poses A and B. Prior to the rmsd calculations, trajectory frames were aligned on the Cα carbons of the GPBAR1 transmembrane helices (TMH).


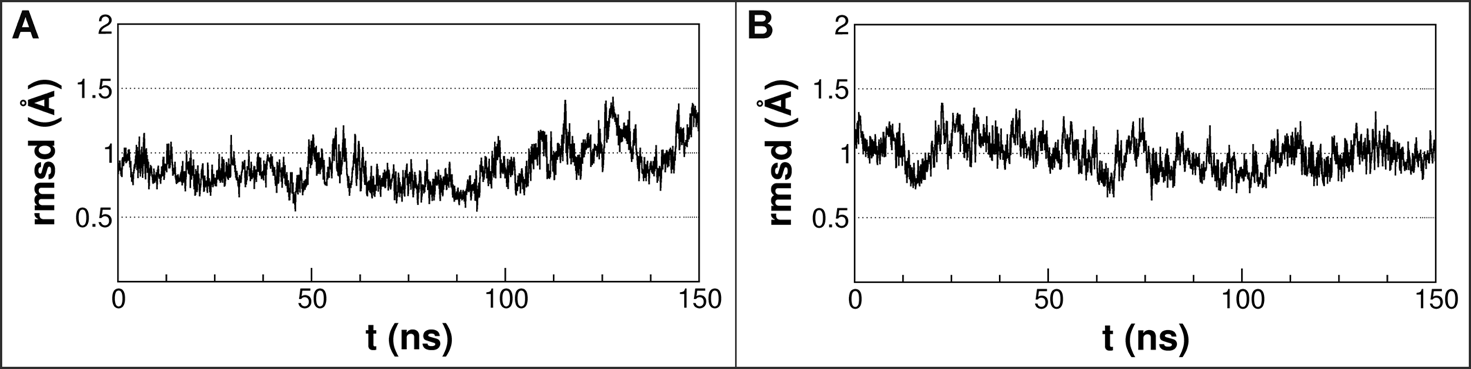


**Supplementary Figure S7.** Average rmsd of the Cα carbons of the GPBAR1 TMHs along the MD simulations on the docking poses A and B. Prior to the rmsd calculations, trajectory frames were aligned on the same atoms.

**Synthetic Procedures for compounds 1-14 and compound F**

**Compound 1**. **(3*R*,5*S*)-5-(3-Phenyl-1,2,4-oxadiazol-5-yl)pyrrolidin-3-ol.** A solution of compound **17** (50 mg, 0.15 mmol) in CH_2_Cl_2_ (1 mL) was treated with trifluoroacetic acid (1 mL) at room temperature for 2 h. The solution was concentrated at reduced pressure to give compound **1**. HPLC purification on a Nucleodur100-5 C18 (5 μm; 4.6 mm i.d. x 250 mm) with MeOH/H_2_O (48:52) as eluent (flow rate 1 mL/min), gave 33 mg of **1** as white solid (quantitative yield, t_R_= 7.5 min). ^1^H NMR (400 MHz, CD_3_OD): δ_H_ 8.11 (2H, dd, *J* = 7.9, 1.6 Hz), 7.58 (1H, ovl), 7.56 (2H, ovl), 5.37 (1H, dd, *J* = 10.7, 7.4 Hz), 4.75 (1H, m), 3.62 (1H, dd, *J* = 12.0, 3.6 Hz), 3.46 (1H, br d, *J* = 12.0 Hz), 2.68 (1H, m), 2.56 (1H, m); ^13^C NMR (100 MHz, CD_3_OD): δ_C_ 176.3, 169.8, 132.7, 130.0 (2C), 128.2 (2C), 127.3, 70.4, 55.1, 53.9, 39.4; HR ESIMS *m/z* 232.1091 [M + H]^+^, C_12_H_14_N_3_O_2_ requires 232.1086.

**Compound 2**. **5-((2*S*,4*S*)-4-Azidopyrrolidin-2-yl)-3-phenyl-1,2,4-oxadiazole.** A solution of compound **18** (40 mg, 0.11 mmol) in CH_2_Cl_2_ (1 mL) was treated with trifluoroacetic acid (1 mL) at room temperature for 2 h. The solution was concentrated at reduced pressure to give compound **2**. HPLC purification on a Nucleodur Sphinx RP (5 μm; 4.6 mm i.d. x 250 mm) with MeOH/H_2_O (40:60) as eluent (flow rate 1 mL/min), gave 25 mg of **2** as white solid (quantitative yield, t_R_= 10.1 min). ^1^H NMR (400 MHz, CD_3_OD): δ_H_ 8.10 (2H, d, *J* = 7.9 Hz), 7.57 (1H, ovl), 7.54 (2H, ovl), 5.21 (1H, dd, *J*=10.7, 7.4 Hz), 4.67 (1H, m), 3.58 (1H, dd, *J*=12.0, 4.9 Hz), 3.48 (1H, br d, *J*=12.0 Hz), 2.95 (1H, m), 2.68 (1H, m); ^13^C NMR (100 MHz, CD_3_OD): δ_C_ 176.9, 169.8, 132.7, 130.2 (2C), 128.2 (2C), 127.6, 60.7, 54.3, 52.6, 35.9; HR ESIMS *m/z* 257.1157 [M + H]^+^, C_12_H_13_N_6_O requires 257.1151.

**Compound 3**. **(3*S*,5*S*)-5-(3-Phenyl-1,2,4-oxadiazol-5-yl)pyrrolidin-3-amine.** A solution of compound **19** (40 mg, 0.121 mmol) in CH_2_Cl_2_ (1 mL) was treated with trifluoroacetic acid (1 mL) at room temperature for 2 h. The solution was concentrated at reduced pressure to give compound **3**. HPLC purification on a Nucleodur Sphinx RP (5 μm; 4.6 mm i.d. x 250 mm) with MeOH/H_2_O (30:70) as eluent (flow rate 1 mL/min), gave 20 mg of compound **3** as white solid (quantitative yield, t_R_= 12.5 min). ^1^H NMR (400 MHz, CD_3_OD): δ_H_ 8.11 (2H, dd, *J*= 8.0, 1.5 Hz); 7.56 (1H, ovl), 7.54 (2H, ovl); 4.69 (1H, dd, *J* = 8.4, 6.5 Hz); 3.92 (1H, m); 3.41 (1H, dd, *J*= 11.6, 6.4 Hz), 3.12 (1H, dd, *J*=11.6, 4.3 Hz); 2.86 (1H, dt, *J*=14.4, 8.4 Hz), 2.28 (1H, dt, *J*=14.4, 6.5 Hz); ^13^C NMR (100 MHz, CD_3_OD): δ_C_ 176.8, 169.8, 132.5, 130.0 (2C), 128.3 (2C), 127.7, 54.0, 52.1, 51.6, 36.1; HR ESIMS *m/z* 221.1249 [M + H]^+^, C_12_H_15_N_4_O requires 221.1246.

**Compound 4**. **1-((3*S*,5*S*)-5-(3-Phenyl-1,2,4-oxadiazol-5-yl)pyrrolidin-3-yl)-3-(4-(trifluoromethyl)phenyl)urea.** To a solution of compound **19** (30 mg, 0.0909 mmol) in CH_2_Cl_2_ (2 mL) was added 4-(trifluoromethyl)phenyl isocyanate (14.3 μL, 0.099 mmol). The reaction mixture was stirred at room temperature for 4 h. The reaction was diluted with CH_2_Cl_2_ and extracted with water (3 x 50 mL), dried with Na_2_SO_4_, filtered and concentrated. Then the solid was treated with TFA (1 mL) in CH_2_Cl_2_ (1 mL). After stirring at room temperature for 2 h, the solvent was evaporated. HPLC purification on a Nucleodur Sphinx RP (5 μm; 4.6 mm i.d. x 250 mm) with MeOH/H_2_O (70:30) as eluent (flow rate 1 mL/min), gave 32 mg of compound **4** as white solid (84% yield over two steps, t_R_= 12.5 min). ^1^H NMR (500 MHz, CD_3_OD): δ_H_ 8.10 (2H, dd, *J* = 7.8, 1.6 Hz), 7.56 (1H, ovl), 7.54 (2H, ovl), 7.53 (4H, ovl), 5.23 (1H, br t, *J* = 8.0 Hz), 4.58 (1H, m), 3.77 (1H, dd, *J*= 11.4, 7.6 Hz), 3.55 (1H, dd, *J*= 11.4, 4.5 Hz), 3.06 (1H, dt, *J*= 13.6, 8.1 Hz), 2.54 (1H, m);^13^C NMR (100 MHz, CD_3_OD): δ_C_ 176.3, 169.5, 157.2, 144.1, 132.5, 129.8 (2C), 128.7, 128.1 (2C), 127.4, 126.8 (2C), 125.1, 119.0 (2C), 54.2, 52.4, 50.9, 36.4; HR ESIMS *m/z* 418.1496 [M + H]^+^, C_20_H_19_N_5_O_2_F_3_ requires 418.1491.

**Compound F**. **1-((3*S*,5*S*)-1-Methyl-5-(3-phenyl-1,2,4-oxadiazol-5-yl)pyrrolidin-3-yl)-3-(4-(trifluoromethyl)phenyl)urea.** Compound **4** (32 mg, 0.062 mmol) was dissolved in 37% aqueous formaldehyde (1 mL) and 95% aqueous HCOOH (1 mL). The reaction was stirred at 80-90 °C overnight. After completion of the reaction, a solution of HCl 6N was added and the mixture was extracted with CH_2_Cl_2_. Then the organic phase was washed with water, dried with Na_2_SO_4_, filtered and concentrated. HPLC purification on a Nucleodur Sphinx RP (5 μm; 4.6 mm i.d. x 250 mm) with MeOH/H_2_O (65:35) with 0.1% TFA as eluent (flow rate 1 mL/min), gave 15 mg of compound **F** as white solid (56%, t_R_= 13.5 min).

^1^H NMR (700 MHz, CD_3_OD): δ_H_ 8.07 (2H, dd, *J* = 7.8, 1.5 Hz), 7.56 (1H, ovl), 7.54 (2H, ovl), 7.53 (4H, ovl), 4.43 (1H, m), 3.88 (1H, dd, *J* = 8.5, 6.8 Hz), 3.09 (1H, d, *J*= 10.5 Hz), 2.85 (1H, m); 2.81 (1H, m), 2.49 (3H, s), 2.23 (1H, m); ^13^C NMR (100 MHz, CD_3_OD): δ_C_ 181.5, 169.5, 157.2, 144.6, 132.4, 129.9 (2C), 128.3, 128.1 (2C), 127.4, 126.8 (2C), 125.1, 119.0 (2C), 63.4, 61.3, 49.9, 40.5, 39.2; HR ESIMS *m/z* 432.1651 [M + H]^+^, requires C_21_H_21_N_5_O_2_F_3_ 432.1647.

**General synthetic procedure for compounds 5-14.** To a solution of compound **19** (0.151 mmol) in CH_2_Cl_2_ (3 mL) were added the required isocyanates (1.2 eq/mol). The reaction mixture was stirred at room temperature for 4 h. The reaction was diluted with CH_2_Cl_2_ and extracted with water (3 x 50 mL), dried with Na_2_SO_4_, filtered and concentrated. Then the solid was treated with TFA (1 mL) in CH_2_Cl_2_ (1 mL). After stirring at room temperature for 2 h, the solvent was evaporated.

**Compound 5**. **1-Phenyl-3-((3*S*,5*S*)-5-(3-phenyl-1,2,4-oxadiazol-5-yl)pyrrolidin-3-yl)urea.** HPLC purification on a Nucleodur Sphinx RP (5 μm; 4.6 mm i.d. x 250 mm) with MeOH/H_2_O (50:50) with 0.1% TFA as eluent (flow rate 1 mL/min), gave compound **5** as white solid in 95% yield (t_R_= 30 min). ^1^H NMR (400 MHz, CD_3_OD): δ_H_ 8.11 (2H, dd, *J* = 7.8, 1.6 Hz), 7.56 (1H, ovl), 7.54 (2H, ovl), 7.34 (2H, d, *J*=7.7 Hz), 7.23 (2H, t, *J*=7.7 Hz), 6.98 (1H, t, *J*=7.7 Hz), 5.26 (1H, t, *J* = 8.7 Hz), 4.58 (1H, m), 3.78 (1H, dd, *J*= 12.2, 7.9 Hz), 3.54 (1H, dd, *J*=12.2, 5.2 Hz), 3.07 (1H, m), 2.53 (1H, m); ^13^C NMR (100 MHz, CD_3_OD): δ_C_ 175.7, 169.7, 157.6, 140.2, 132.7, 130.0 (2C), 129.6 (2C),128.4 (2C), 127.1, 123.7, 120.4 (2C) 54.6, 52.1, 50.5, 35.6; HR ESIMS *m/z* 350.1621 [M + H]^+^, C_19_H_20_N_5_O_2_ requires 350.1617.

**Compound 6**. **1-((3*S*,5*S*)-5-(3-phenyl-1,2,4-oxadiazol-5-yl)pyrrolidin-3-yl)-3-(p-tolyl)urea.** HPLC purification on a Nucleodur Sphinx RP (5 μm; 4.6 mm i.d. x 250 mm) with MeOH/H_2_O (50:50) with 0.1% TFA as eluent (flow rate 1 mL/min), gave compound **6** as white solid in 86% yield (t_R_= 33 min). ^1^H NMR (400 MHz, CD_3_OD): δ_H_ 8.12 (2H, dd, *J* = 7.8, 1.5 Hz), 7.56 (1H, ovl), 7.54 (2H, ovl), 7.21 (2H, d, *J*= 8.3 Hz), 7.06 (2H, d, *J*= 8.3 Hz), 5.26 (1H, t, *J* = 8.4 Hz), 4.58 (1H, m), 3.78 (1H, dd, *J*= 12.1, 7.7 Hz), 3.56 (1H, dd, *J*= 12.1, 5.0 Hz), 3.07 (1H, dt, *J*= 13.6, 8.4 Hz), 2.53 (1H, m), 2.26 (3H, s); ^13^C NMR (100 MHz, CD_3_OD): δ_C_ 176.3, 169.6, 157.5, 137.4, 133.5, 132.6, 130.0 (4C), 128.1 (2C), 126.9, 120.6 (2C), 54.4, 52.1, 50.4, 35.6, 20.4; HR ESIMS *m/z* 364.1776 [M + H]^+^, C_20_H_22_N_5_O_2_requires 364.1773.

**Compound 7**. **1-(4-Methoxyphenyl)-3-((3*S*,5*S*)-5-(3-phenyl-1,2,4-oxadiazol-5-yl)pyrrolidin-3-yl)urea.** HPLC purification on a Nucleodur Sphinx RP (5μm; 4.6 mm i.d. x 250 mm) with MeOH/H_2_O (50:50) with 0.1% TFA as eluent (flow rate 1 mL/min), gave compound **7** as white solid in 79% yield (t_R_= 14.1 min). ^1^H NMR (400 MHz, CD_3_OD): δ_H_ 8.11 (2H, dd, *J* = 7.8, 1.5 Hz), 7.58 (1H, ovl), 7.55 (2H, ovl), 7.21 (2H, d, *J*= 8.8 Hz), 6.83 (2H, d, *J*= 8.8 Hz), 5.24 (1H, t, *J* = 8.4 Hz), 4.56 (1H, m), 3.74 (1H, ovl), 3.73 (3H, s), 3.54 (1H, dd, *J*= 12.0, 4.9 Hz), 3.04 (1H, m), 2.51 (1H, m); ^13^C NMR (100 MHz, CD_3_OD): δ_C_ 175.4, 169.5, 157.8, 157.0, 132.6, 132.3, 130.0 (2C), 128.2 (2C), 126.8, 122.8 (2C), 114.8 (2C), 55.5, 54.5, 52.1, 50.4, 35.5; HR ESIMS *m/z* 380.1727 [M + H]^+^, C_20_H_22_N_5_O_3_ requires 380.1723.

**Compound 8**. **1-(4-Fluorophenyl)-3-((3*S*,5*S*)-5-(3-phenyl-1,2,4-oxadiazol-5-yl)pyrrolidin-3-yl)urea.** HPLC purification on a Nucleodur Sphinx RP (5μm; 4.6 mm i.d. x 250 mm) with MeOH/H_2_O (50:50) with 0.1% TFA as eluent (flow rate 1 mL/min), gave compound **8** as white solid in 93% yield (t_R_= 17.4 min). ^1^H NMR (400 MHz, CD_3_OD): δ_H_ 8.11 (2H, dd, *J* = 7.8, 1.5 Hz), 7.58 (1H, ovl), 7.55 (2H, ovl), 7.33 (2H, dd, *J*= 8.9,4.7 Hz), 6.98 (2H, t, *J*= 8.9 Hz), 5.27 (1H, t, *J* = 8.6 Hz), 4.58 (1H, m), 3.78 (1H, dd, *J*= 11.5, 7.7 Hz), 3.54 (1H, dd, *J*= 11.5, 4.7 Hz), 3.06 (1H, dt, *J*= 13.6, 7.8 Hz), 2.55 (1H, m); ^13^C NMR (100 MHz, CD_3_OD): δ_C_ 175.4, 169.6, 159.8, 157.5, 136.2, 132.7, 130.0 (2C), 128.2 (2C), 126.9, 122.1 (2C), 115.9 (2C), 54.5, 51.9, 50.3, 35.3; HR ESIMS *m/z* 368.1527 [M + H]^+^, C_19_H_19_N_5_O_2_F requires 368.1523.

**Compound 9**. **1-(4-Chlorophenyl)-3-((3*S*,5*S*)-5-(3-phenyl-1,2,4-oxadiazol-5-yl)pyrrolidin-3-yl)urea.** HPLC purification on a Nucleodur Sphinx RP (5μm; 4.6 mm i.d. x 250 mm) with MeOH/H_2_O (58:42) with 0.1% TFA as eluent (flow rate 1 mL/min), gave compound **9** as white solid in 78% yield (t_R_= 13.5 min). ^1^H NMR (700 MHz, CD_3_OD): δ_H_ 8.11 (2H, d, *J* = 8.0 Hz), 7.57 (1H, ovl), 7.54 (2H, ovl), 7.33 (2H, d, *J*= 7.2 Hz), 7.21 (2H, d, *J*= 7.2 Hz), 5.26 (1H, t, *J* = 8.7 Hz), 4.56 (1H, m), 3.75 (1H, dd, *J*= 11.7, 7.8 Hz), 3.54 (1H, dd, *J*= 11.7, 4.7 Hz), 3.06 (1H, dt, *J* = 13.7, 8.1 Hz), 2.50 (1H, m); ^13^C NMR (175 MHz, CD_3_OD): δ_C_ 175.5, 169.6, 157.6, 139.0, 132.7, 129.9 (2C), 129.5 (2C), 128.3 (3C), 126.9, 121.3 (2C), 54.5, 52.0, 50.4, 35.3; HR ESIMS *m/z* 384.1229 [M + H]^+^, C_19_H_19_N_5_O_2_Cl requires 384.1227.

**Compound 10**. **1-Cyclohexyl-3-((3*S*,5*S*)-5-(3-phenyl-1,2,4-oxadiazol-5-yl)pyrrolidin-3-yl)urea.** HPLC purification on a Nucleodur Sphinx RP (5μm; 4.6 mm i.d. x 250 mm) with MeOH/H_2_O (55:45) with 0.1% TFA as eluent (flow rate 1 mL/min), gave compound **10** as white solid in quantitative yield (t_R_= 13.5 min). ^1^H NMR (500 MHz, CD_3_OD): δ_H_ 8.11 (2H, dd, *J* = 7.9, 1.5 Hz), 7.58 (1H, ovl), 7.55 (2H, ovl), 5.22 (1H, t, *J*= 7.6 Hz), 4.48 (1H, m), 3.72 (1H, m), 3.48 (1H, ovl), 3.43 (1H, ovl), 3.02 (1H, dt, *J*= 13.5, 7.9 Hz), 2.45 (1H, m), 1.83 (2H, m), 1.70 (2H, m), 1.58 (1H, m), 1.31 (2H, m), 1.17 (1H, m), 1.13 (2H, m); ^13^C NMR (100 MHz, CD_3_OD): δ_C_ 175.7, 169.9, 159.6, 132.7, 130.1 (2C), 128.3 (2C), 127.0, 54.5, 52.3, 50.4, 49.8, 35.6, 34.4 (2C), 26.4, 25.1 (2C); HR ESIMS *m/z* 386.2091 [M + H]^+^, C_19_H_26_N_5_O_2_ requires 356.2087.

**Compound 11**. **1-Benzyl-3-((3*S*,5*S*)-5-(3-phenyl-1,2,4-oxadiazol-5-yl)pyrrolidin-3-yl)urea.** HPLC purification on a Nucleodur Sphinx RP (5μm; 4.6 mm i.d. x 250 mm) with MeOH/H_2_O (50:50) with 0.1% TFA as eluent (flow rate 1 mL/min), gave compound **11** as white solid in 91% yield (t_R_= 17.4 min). ^1^H NMR (400 MHz, CD_3_OD): δ_H_ 8.11 (2H, dd, *J*= 7.8, 1.5 Hz), 7.58 (1H, ovl), 7.54 (2H, ovl), 7.27 (2H, ovl), 7.25 (1H, ovl), 7.22 (2H, ovl), 5.21 (1H, t, *J*= 7.6 Hz), 4.50 (1H, m), 4.28 (2H, s), 3.72 (1H, dd, *J* = 11.9, 7.6 Hz), 3.48 (1H, dd, *J*= 11.9, 4.9 Hz), 3.02 (1H, dt, *J*= 13.6, 8.0 Hz), 2.44 (1H, m); ^13^C NMR (100 MHz, CD_3_OD): δ_C_ 175.8, 169.8, 160.2, 140.8, 132.7, 130.0 (2C), 129.2 (2C), 128.3 (2C), 127.9 (3C), 127.2, 54.6, 52.2, 50.4, 44.4, 35.5; HR ESIMS *m/z* 364.1782 [M + H]^+^, C_20_H_22_N_5_O_2_ requires 364.1773.

**Compound 12**. **1-(4-Phenoxyphenyl)-3-((3*S*,5*S*)-5-(3-phenyl-1,2,4-oxadiazol-5-yl)pyrrolidin-3-yl)urea.** HPLC purification on a Nucleodur Sphinx RP (5μm; 4.6 mm i.d. x 250 mm) with MeOH/H_2_O (57:43) with 0.1% TFA as eluent (flow rate 1 mL/min), gave compound **12** as white solid in 82% yield (t_R_= 10.8 min). ^1^H NMR (500 MHz, CD_3_OD): δ_H_ 8.11 (2H, d, *J* = 8.2 Hz), 7.58 (1H, ovl), 7.54 (2H, ovl), 7.32 (2H, d, *J* = 8.8 Hz), 7.31 (2H, t, *J* = 7.4 Hz), 7.08 (1H, t, *J* = 7.4 Hz), 6.93 (2H, d, *J* = 7.4 Hz), 6.90 (2H, d, *J* = 8.8 Hz), 5.26 (1H, t, *J* = 8.9 Hz), 4.58 (1H, m), 3.78 (1H, dd, *J* = 12.0, 7.5 Hz), 3.58 (1H, dd, *J* = 12.0, 5.2 Hz), 3.07 (1H, m), 2.54 (1H, m);^13^C NMR (125 MHz, CD_3_OD): δ_C_ 175.7, 169.6, 159.3, 157.7,153.9, 135.8, 132.9, 130.7 (2C), 129.9 (2C), 128.3 (2C), 127.2, 123.8, 122.4 (2C), 120.3 (2C), 118.9 (2C), 54.6, 52.0, 50.4, 35.4; HR ESIMS *m/z* 442.1882 [M + H]^+^, C_25_H_24_N_5_O_3_ requires 442.1879.

**Compound 13**. **1-(Naphthalen-1-yl)-3-((3*S*,5*S*)-5-(3-phenyl-1,2,4-oxadiazol-5-yl)pyrrolidin-3-yl)urea.**

HPLC purification on a Nucleodur Sphinx RP (5μm; 4.6 mm i.d. x 250 mm) with MeOH/H_2_O (55:45) with 0.1% TFA as eluent (flow rate 1 mL/min), gave compound **13** as white solid in 74% yield (t_R_= 24 min). ^1^H NMR (400 MHz, CD_3_OD): δ_H_ 8.11 (2H, dd, *J* = 7.6, 1.6 Hz), 7.97 (1H, m), 7.86 (1H, m), 7.69 (1H, d, *J*= 8.2 Hz), 7.63 (1H, d, *J*= 7.4 Hz), 7.58 (1H, ovl), 7.54 (2H, ovl), 7.50 (2H, m), 7.42 (1H, t, *J*= 7.9 Hz), 5.26 (1H, t, *J* = 8.5 Hz), 4.61 (1H, m), 3.79 (1H, dd, *J*= 11.5, 7.6 Hz), 3.60 (1H, dd, *J*= 11.5, 4.9 Hz), 3.07 (1H, dt, *J*= 13.6, 8.9 Hz), 2.58 (1H, m); ^13^C NMR (100 MHz, CD_3_OD): δ_C_ 175.6, 169.6, 158.5, 135.6, 134.4, 132.7, 129.9 (2C), 129.4, 128.5 (2C), 127.0, 126.8 (2C), 126.4, 126.2, 124.7, 122.3, 122.1, 54.5, 52.2, 50.6, 35.8; HR ESIMS *m/z* 400.1778 [M + H]^+^, C_23_H_22_N_5_O_2_ requires 400.1773.

**Compound 14**. **1-([1,1'-Biphenyl]-4-yl)-3-((3*S*,5*S*)-5-(3-phenyl-1,2,4-oxadiazol-5-yl)pyrrolidin-3-yl)urea.**

HPLC purification on a Nucleodur Sphinx RP (5 μm; 4.6 mm i.d. x 250 mm) with MeOH/H_2_O (65:35) with 0.1% TFA as eluent (flow rate 1 mL/min), gave compound **14** as white solid in 93% yield (t_R_= 22.5 min). ^1^H NMR (500 MHz, CD_3_OD): δ_H_8.11 (2H, dd, *J* = 7.7, 1.4 Hz), 7.58 (1H, ovl), 7.55 (2H, ovl), 7.54 (2H, ovl), 7.52 (2H, d, *J*= 8.7 Hz), 7.43 (2H, d, *J*= 8.7 Hz), 7.39 (2H, t, *J* = 7.5 Hz), 7.29 (1H, t, *J*= 7.5 Hz), 5.27 (1H, t, *J* = 8.4 Hz), 4.60 (1H, m), 3.79 (1H, dd, *J*= 12.0, 7.5 Hz), 3.59 (1H, dd, *J* = 12.0, 5.2 Hz), 3.09 (1H, dt, *J* = 13.8, 7.9 Hz), 2.56 (1H, m); ^13^C NMR (100 MHz, CD_3_OD): δ_C_ 175.4, 169.7, 157.8, 142.0, 140.0, 137.1, 132.6, 129.9 (2C), 129.6 (2C), 128.3 (2C), 128.2 (2C), 127.7, 127.4 (2C), 127.0, 120.5 (2C), 54.4, 52.1, 50.4, 35.6; HR ESIMS *m/z* 426.1933 [M + H]^+^, C_25_H_24_N_5_O_2_ requires 426.1930.

**Compound 16. N-hydroxybenzamidine**. To a solution of benzonitrile (**15**) (1 mL, 9.70 mmol) in dry methanol (5 mL), potassium carbonate (2 g, 14.6 mmol) and hydroxylamine hydrochloride (1.7 g, 24.2 mmol) were added, and the mixture was stirred at reflux for 5 h. The resulting solution was then concentrated under vacuum, diluted with water and extracted with CH_2_Cl_2_ (3 x 20 mL). The organic phases were dried (Na_2_SO_4_), filtered and concentrated *in vacuo* to give 1.3 g of compound **16** (quantitative yield), that was subjected to next step without any purification. ^1^H NMR (400 MHz, CDCl_3_): δ_H_ 7.55 (2H, d, *J*= 7.9 Hz), 7.34 (3H, ovl), 4.88 (2H, s, NH_2_). ^13^C NMR (100 MHz, CDCl_3_): δ_C_ 152.5, 130.0 (2C), 129.1, 128.7 (2C), 126.0; HR ESIMS *m/z* 137.0721 [M + H]^+^, C_7_H_9_ON_2_ requires 137.0715.

**Compound 17**. **(2*S*,4*R*)-tert-Butyl-4-Hydroxy-2-(3-phenyl)-1,2,4-oxadiazol-5-yl)pyrrolidine-1-carboxylate.** DIPEA (1.4 mL, 7.9 mmol) was added to a solution of compound **16** (600 mg, 4.41 mmol) and (3*R*)-hydroxy-Boc-L-proline (1.1 g, 5.3 mmol) dissolved in dry DMF (5 mL). HBTU (2.7 g, 6.6 mmol) was then added to the mixture at room temperature. The mixture was stirred vigorously at 80°C for 12 h then partitioned between water and ethyl acetate (20 mL). The organic layer was collected and washed twice with a saturated LiBr solution, then with saturated NaHCO_3_ solution and brine, dried over Na_2_SO_4_, filtered and concentrated under reduced pressure. The resulted residue was purified on silica column using hexane and ethyl acetate 7:3, gave 880 mg of compound **17** (60% yield). ^1^H NMR (400 MHz, CD_3_OD): δ_H_ 8.04 (2H, d, *J* = 7.8 Hz), 7.53 (2H, ovl), 7.51 (1H, ovl), 5.21 (1H, t, *J*=7.9 Hz), 4.53 (1H, m), 3.72 (1H, dd, *J*=11.6, 4.1 Hz), 3.58 (1H, br d, *J*=11.6 Hz), 2.44 (1H, m), 2.28 (1H, m), 1.45 (9H, s); ^13^C NMR (100 MHz, CD_3_OD): δ_C_ 182.4, 169.7, 155.5, 132.6, 130.2 (2C), 128.3 (2C), 127.3, 82.1, 69.9, 55.7,54.0, 41.8, 28.3 (3C); HR ESIMS *m/z* 332.1613[M + H]^+^, C_17_H_22_N_3_O_4_ requires 332.1610.

**Compound 18**. **(2*S*,4*S*)-tert-Butyl 4-azido-2-(3-phenyl-1,2,4-oxadiazol-5-yl)pyrrolidine-1-carboxylate**. To a solution of compound **17** (800 mg, 2.41 mmol) in dry ethyl ether (3 mL), mesyl chloride (930 μL, 12.1 mmol) and TEA (2.1 mL, 14.5 mmol) was added, and the mixture was stirred at -10 °C for 1 h. It was poured into saturated NaHCO_3_ solution (10 mL) and extracted with ethyl ether (3 × 20 mL). The combined organic layer was washed with water (20 mL), and then dried over anhydrous Na_2_SO_4_ and evaporated *in vacuo* to give 980 mg of mesyloxy derivative (quantitative yield), that was subjected to next step without any purification. The intermediate (980 mg, 2.40 mmol) was dissolved in dry DMSO (5 mL) and sodium azide (1.6 g, 24 mmol) was added. The mixture was stirred vigorously at 150 °C for 12 h then partitioned between water and ethyl acetate (10 mL). The organic phases were dried (Na_2_SO_4_), filtered and concentrated *in vacuo* to give 640 mg of compound **18** (75% yield). ^1^H NMR (400 MHz, CD_3_OD): δ_H_ 8.06 (2H, d, *J* = 7.8 Hz), 7.53 (2H, ovl), 7.52 (1H, ovl), 5.25 (1H, m), 4.43 (1H, m), 3.78 (1H, dd, *J*=11.6, 4.1 Hz), 3.50 (1H, br d, *J*=11.6 Hz), 2.76 (1H, m), 2.39 (1H, m), 1.50 (9H, s); HR ESIMS *m/z* 357.1678 [M + H]^+^, C_17_H_21_N_6_O_3_ requires 357.1675.

**Compound 19**. **(2*S*,4*S*)-tert-Butyl 4-amino-2-(3-phenyl-1,2,4-oxadiazol-5-yl)pyrrolidine-1-carboxylate.** To a solution of **18** (600 mg, 1.69 mmol) in methanol (5 mL) and water (500 μL) were added solid ammonium chloride (895 mg, 16.9 mmol) and zinc powder (550 mg, 8.45 mmol). After stirring overnight, the reaction mixture was filtered through a short pad of Celite and concentrated. The resulting residue was diluted with ethyl acetate, washed with brine, dried over Na_2_SO_4_ anhydrous and evaporated under reduced pressure to give 340 mg of compound **19** (61% yield). ^1^H NMR (400 MHz, CD_3_OD): δ_H_ 8.04 (2H, d, *J* = 7.9 Hz), 7.53 (2H, ovl), 7.51 (1H, ovl), 5.14 (1H, t, *J*=7.5 Hz), 3.86 (1H, m), 3.67 (1H, m), 3.38 (1H, dd, *J*=10.6, 6.4 Hz), 2.75 (1H, m), 2.05 (1H, m), 1.44 (9H, s); HR ESIMS *m/z* 331.1775 [M + H]^+^, C_17_H_23_N_4_O_3_ requires 331.1770.


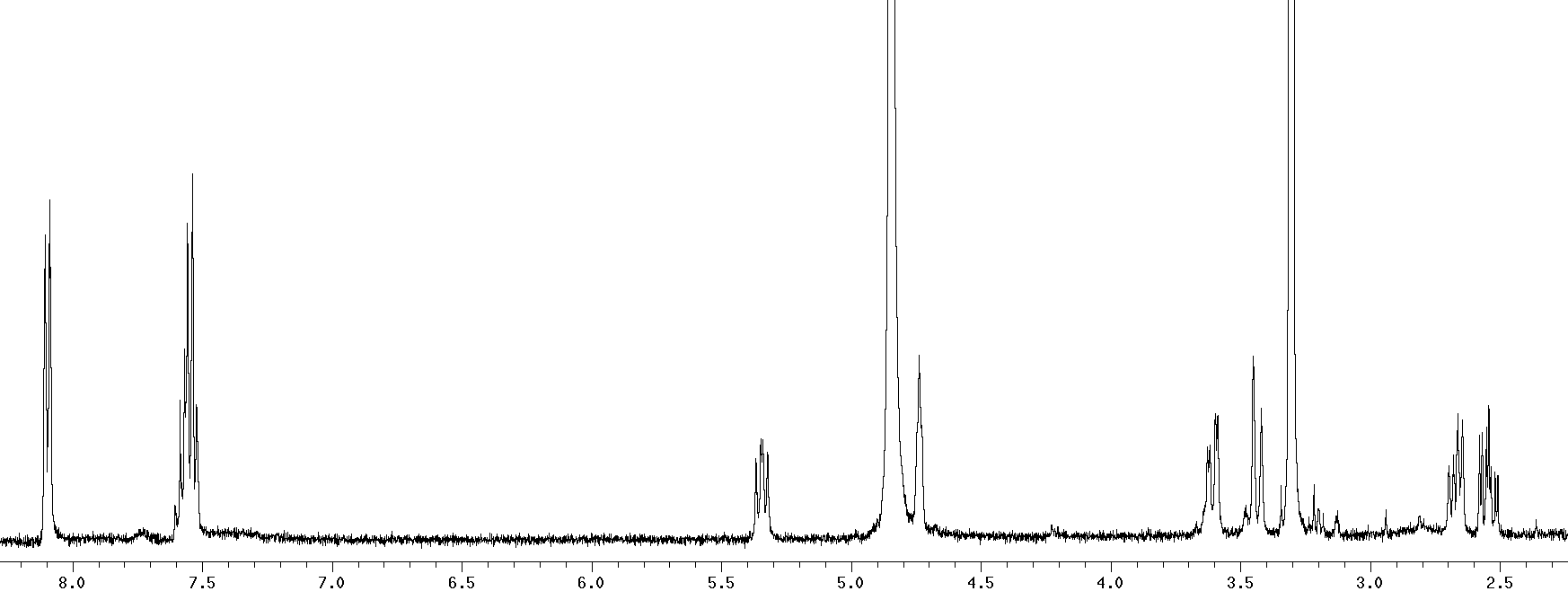
 **Supplementary Figure S8**.^1^H NMR (400 MHz, CD_3_OD) of compound **1**


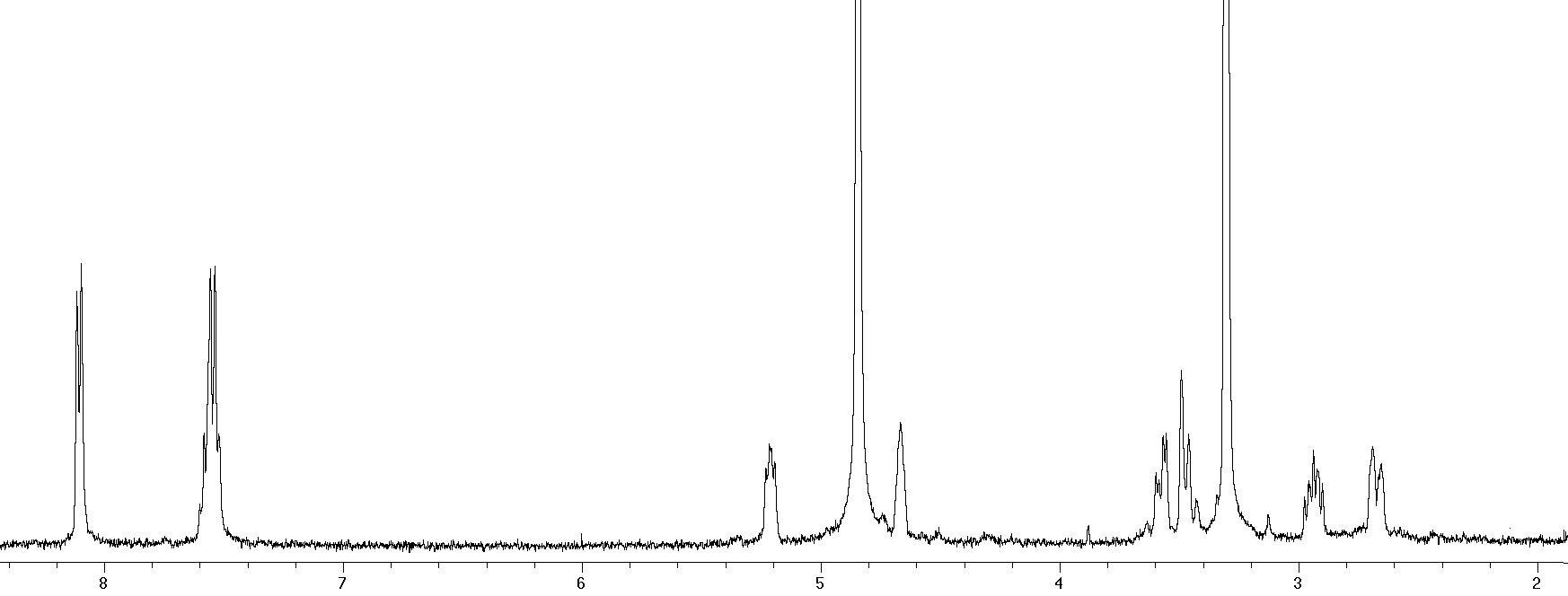


**Supplementary Figure S9**.^1^H NMR (400 MHz, CD_3_OD) of compound **2**

 **Supplementary Figure S10**.^1^H NMR (400 MHz, CD_3_OD) of compound **3**

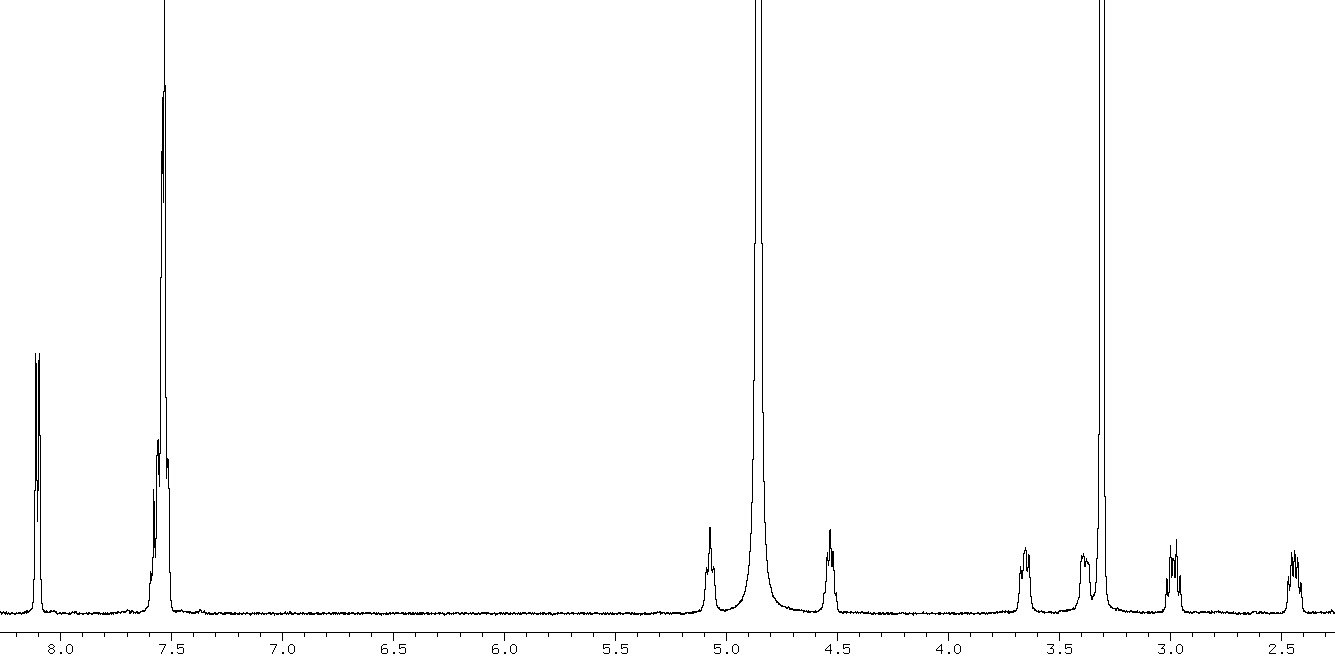


**Supplementary Figure S11**. ^1^H NMR (500 MHz, CD_3_OD) of compound **4**

**Supplementary Figure S12**.^1^H NMR (400 MHz, CD_3_OD) of compound **5**

^^

**Supplementary Figure S13**.^1^H NMR (400 MHz, CD_3_OD) of compound **6**

**Supplementary Figure S14**.^1^H NMR (400 MHz, CD_3_OD) of compound **7**


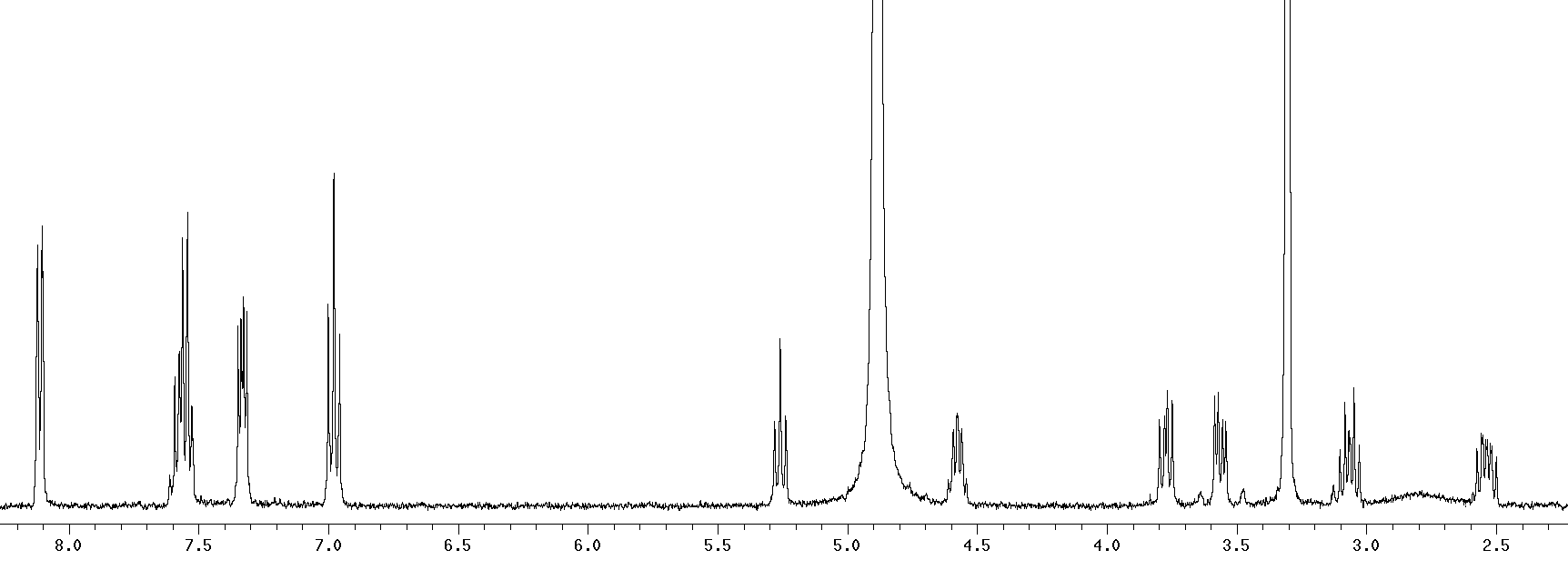


**Supplementary Figure S15**.^1^H NMR (400 MHz, CD_3_OD) of compound **8**


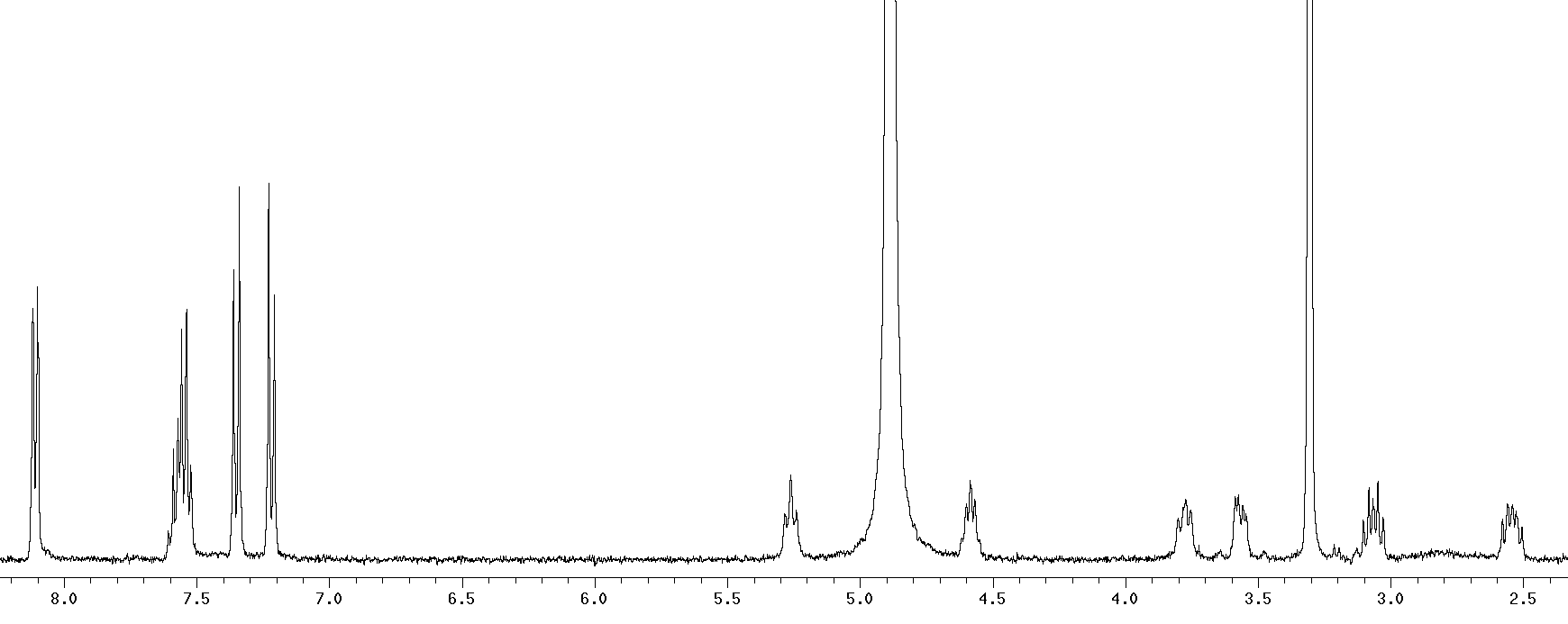


**Supplementary Figure S16**.^1^H NMR (400 MHz, CD_3_OD) of compound **9**


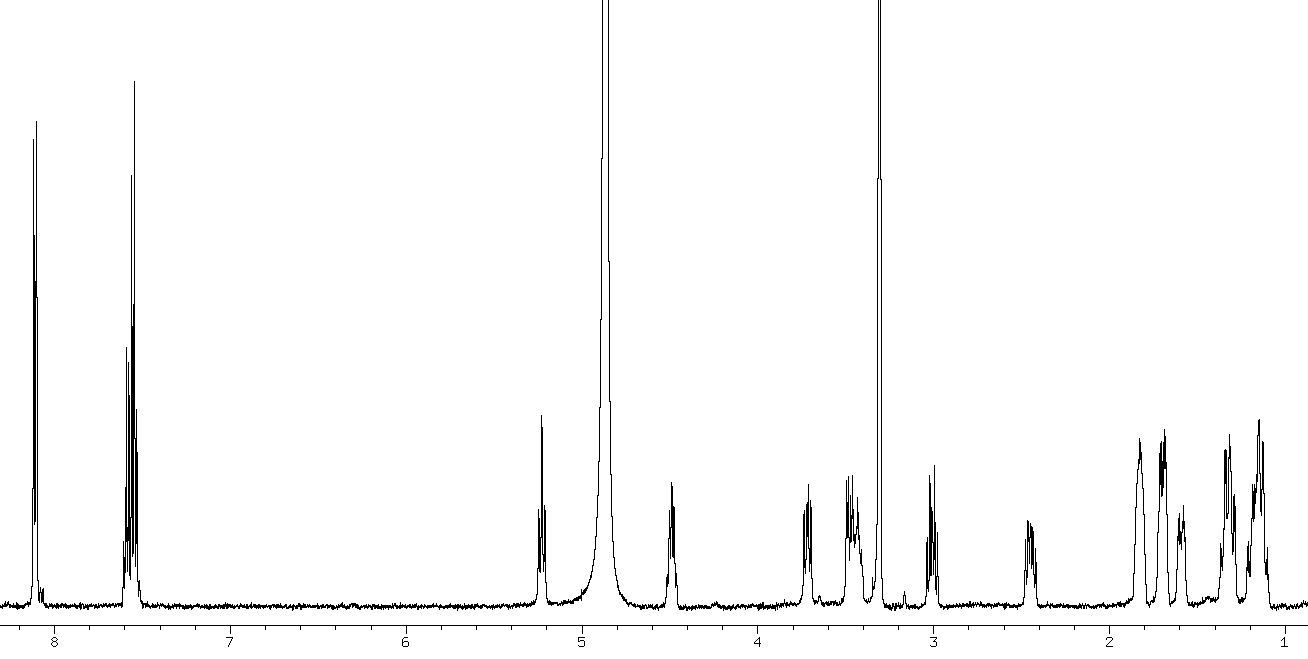


**Supplementary Figure S17**.^1^H NMR (500 MHz, CD_3_OD) of compound **10**

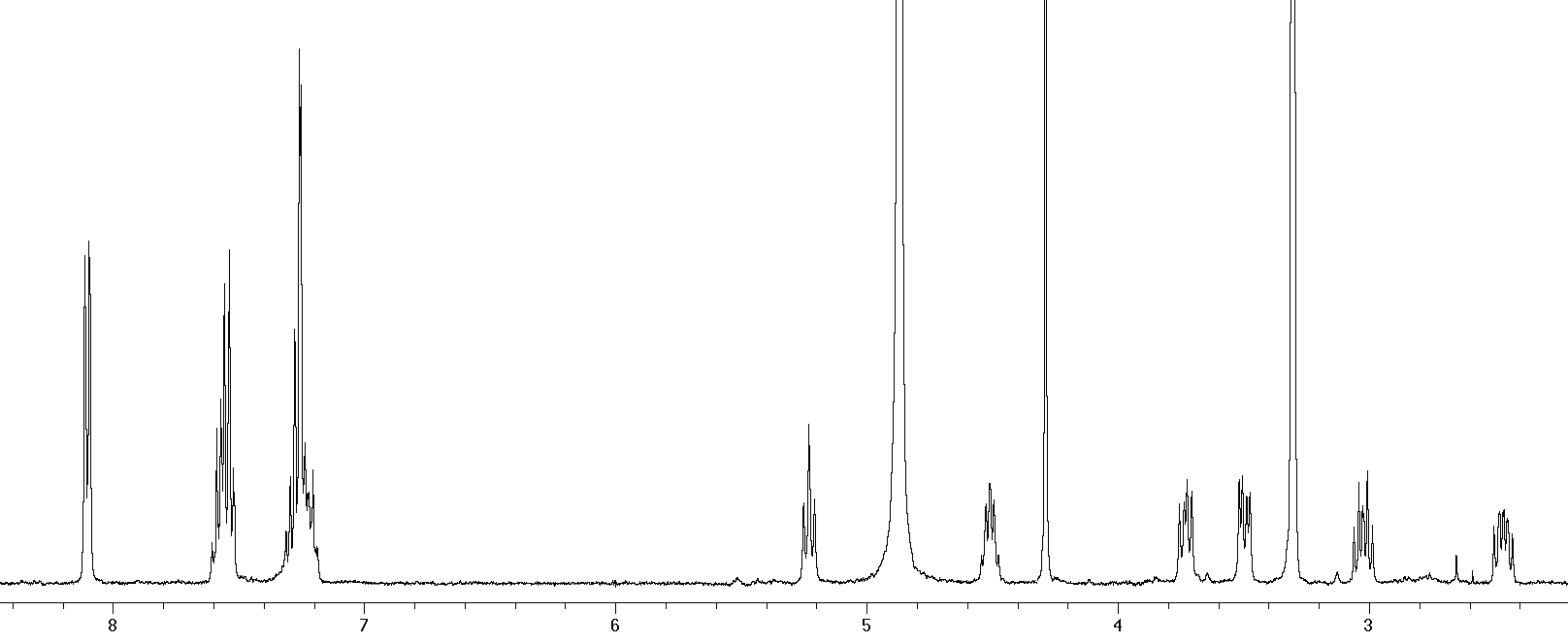


**Supplementary Figure S18**.^1^H NMR (400 MHz, CD_3_OD) of compound **11**


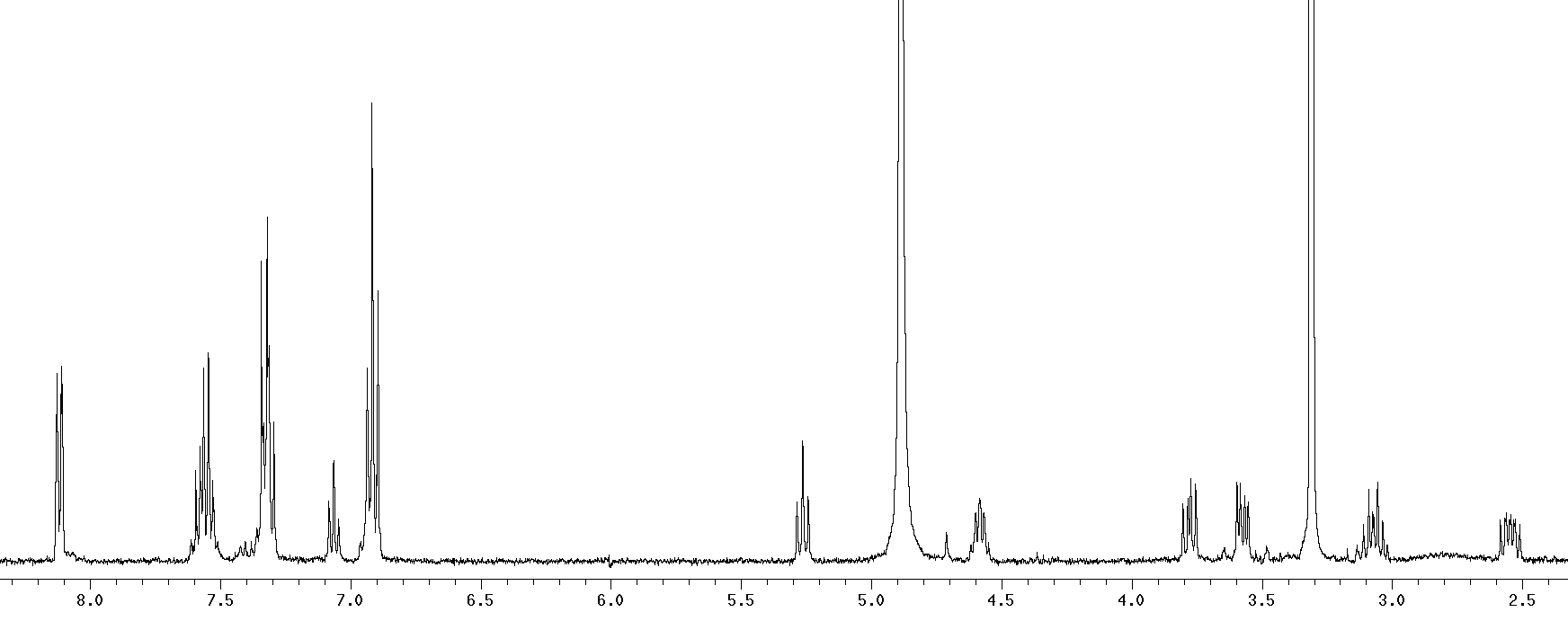


**Supplementary Figure S19**.^1^H NMR (400 MHz, CD_3_OD) of compound **12**


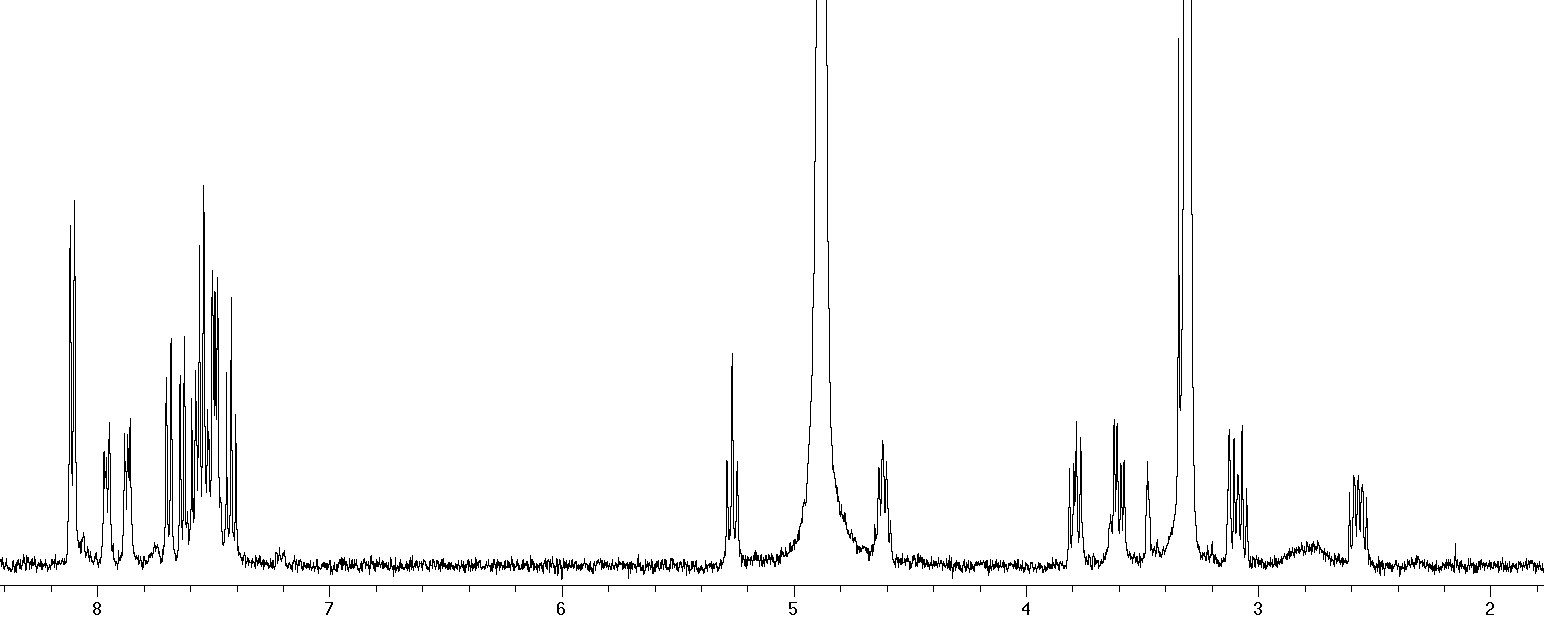


**Supplementary Figure S20**.^1^H NMR (400 MHz, CD_3_OD) of compound **13**


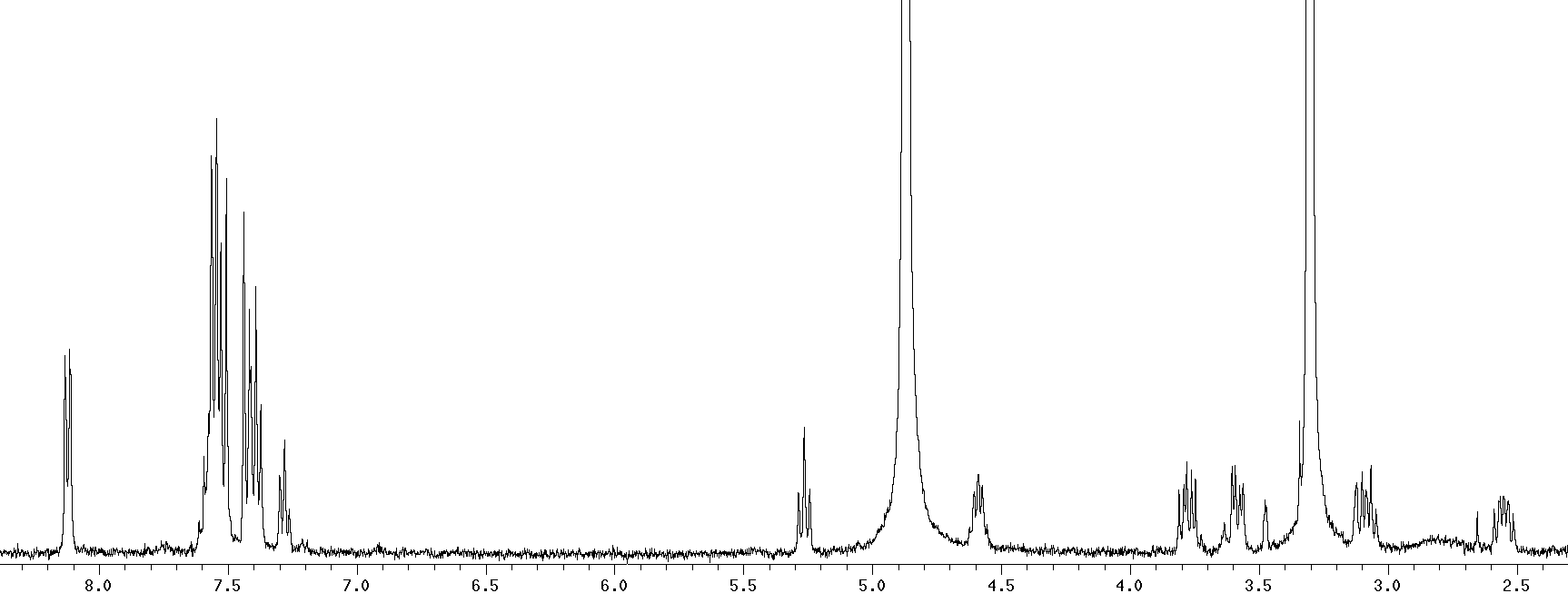
 **Supplementary Figure S21**.^1^H NMR (500 MHz, CD_3_OD) of compound **14**
